# The Long History of Vitamin C: From Prevention of the Common Cold to Potential Aid in the Treatment of COVID-19

**DOI:** 10.3389/fimmu.2020.574029

**Published:** 2020-10-28

**Authors:** Giuseppe Cerullo, Massimo Negro, Mauro Parimbelli, Michela Pecoraro, Simone Perna, Giorgio Liguori, Mariangela Rondanelli, Hellas Cena, Giuseppe D’Antona

**Affiliations:** ^1^Department of Movement Sciences and Wellbeing, University of Naples Parthenope, Naples, Italy; ^2^Centro di Ricerca Interdipartimentale nelle Attività Motorie e Sportive (CRIAMS)—Sport Medicine Centre, University of Pavia, Voghera, Italy; ^3^Department of Pharmacy, University of Salerno, Fisciano, Italy; ^4^Department of Biology, College of Science, University of Bahrain, Sakhir, Bahrain; ^5^IRCCS Mondino Foundation, Pavia, Italy; ^6^Department of Public Health, Experimental and Forensic Medicine, University of Pavia, Pavia, Italy; ^7^Clinical Nutrition and Dietetics Service, Unit of Internal Medicine and Endocrinology, ICS Maugeri IRCCS, University of Pavia, Pavia, Italy

**Keywords:** vitamin C supplementation, viral infections, COVID-19, pneumonia, immune function, athletes, non-communicable diseases, frail elderly subjects

## Abstract

From Pauling’s theories to the present, considerable understanding has been acquired of both the physiological role of vitamin C and of the impact of vitamin C supplementation on the health. Although it is well known that a balanced diet which satisfies the daily intake of vitamin C positively affects the immune system and reduces susceptibility to infections, available data do not support the theory that oral vitamin C supplements boost immunity. No current clinical recommendations support the possibility of significantly decreasing the risk of respiratory infections by using high-dose supplements of vitamin C in a well-nourished general population. Only in restricted subgroups (e.g., athletes or the military) and in subjects with a low plasma vitamin C concentration a supplementation may be justified. Furthermore, in categories at high risk of infection (i.e., the obese, diabetics, the elderly, etc.), a vitamin C supplementation can modulate inflammation, with potential positive effects on immune response to infections. The impact of an extra oral intake of vitamin C on the duration of a cold and the prevention or treatment of pneumonia is still questioned, while, based on critical illness studies, vitamin C infusion has recently been hypothesized as a treatment for COVID-19 hospitalized patients. In this review, we focused on the effects of vitamin C on immune function, summarizing the most relevant studies from the prevention and treatment of common respiratory diseases to the use of vitamin C in critical illness conditions, with the aim of clarifying its potential application during an acute SARS-CoV2 infection.

## Introduction

Vitamin C (ascorbic acid) plays an important role in the normal functioning of the immune system (1–4) and its use in preventing and/or treating infections has strongly attracted the interest of physicians and investigators for almost a century. A plethora of papers have been published on this topic, but, although it is well known that a deficiency of vitamin C due to a low nutritional intake leads to a greater susceptibility to infections (5), the possibility of reducing the incidence of viral diseases in a well-nourished population through the use of dietary supplements based on vitamin C is not adequately supported in literature. At present, very little evidence supports the benefit of high doses of vitamin C supplementation on immune function in healthy individuals (4, 6) and several authors have underlined that this practice is ineffective in preventing the common cold and viral infections in most subjects (7–12). Despite this, the popular belief that an extra intake of vitamin C can boost the immune system is still widespread and every year the market claims that the use of supplements is a winter remedy to prevent infectious disease.

While scientists’ current position is not to recommend the use of vitamin C supplementation to prevent viral invasions in healthy subjects, more promising—though questioned—data seem instead to emerge from the intravenous administration (IA) of this vitamin in acute respiratory conditions or critical illnesses. Furthermore a potential pharmacological role during early phases of the new coronavirus (SARS-CoV2) infection and its related disease (COVID-19) was recently put forward (13–18). The rapid worldwide spread of SARS-CoV2 and the consequent pandemic emergency recognized by World Health Organization urgently requires a global effort to identify anything that can treat symptoms and reduce deaths. At the beginning of June, more than 6,600,000 cases of infection and 391,000 deaths related to COVID-19 had been reported globally and the number of cases is constantly increasing worldwide (19). Currently, no specific antiviral therapy has been approved for the cure of COVID-19 (20), and this has led researchers to speculate on a possible adjuvant treatment based on indirect evidence from severely ill patients and patients with sepsis conditions (21). Sepsis is a life-threatening organ dysfunction caused by an impaired host response to infection, characterized by a dramatic failure of the circulatory, metabolic, and immune systems, and recognized as the primary cause of death from infection: patients who develop septic shock can have hospital mortality rates of up to 50% (22). On these clinical conditions literature shows that high doses of vitamin C by intravenous infusion may reduce the inflammatory cytokine-related production and potentially improve important outcomes such as the duration of mechanical ventilation time and mortality rates (13–18). This is of particular importance since acute respiratory distress syndrome (ARDS) is one of the most frequent severe conditions registered in COVID-19 patients (23). ARDS is a serious and, in some cases fatal, syndrome characterized by a strong inflammatory response with massive alveolar damage and multiple organ failure, requiring intensive care unit (ICU) treatment (24). Authors reported a percentage of ARDS cases of around 15% among hospitalized patients with SARS-CoV2 infection (25).

Data on the use of intravenous-administered vitamin C in COVID-19 patients are still unavailable, but clinical trials to explore the efficacy of this treatment are currently in progress in several countries (26) and important results will be available soon.

Based on the above, the aim of this review is firstly to summarize the immunological role of vitamin C with a description of its potential effects as a dietary supplement, on the mechanisms involved during respiratory viral infections, and in relation to the inflammatory response considering different subject categories and clinical conditions; secondly, the manuscript describes the updated literature on the IA of vitamin C in the treatment of severe sepsis and ARDS conditions, with the aim of establishing whether the current clinical background on this topic offers strong enough perspectives to propose vitamin C for a pharmacological application to reduce the dramatic cytokine production and regulate other recognized COVID-19-related immune responses.

## Physiology of Vitamin C

### Bioavailability

Vitamin C is an essential nutrient that must be taken through the diet as humans are unable to synthesize it (27). Thus, our body has developed an effective adaptation system which maintains the organic reserves of vitamin C and prevents its deficiency due to a low dietary intake. These adaptations include a higher absorption and recycling capacity of vitamin C compared to other animal species (e.g., goat and reptiles), which can normally produce it (28, 29). Animal studies have shown that vitamin C is preferentially stored in the brain, adrenal gland, liver and lungs (30–33) but its levels in these organs are rapidly depleted after about one week of dietary insufficiency (31). In humans, skeletal muscle represents the major pool of vitamin C (34). Muscle fibers also lose vitamin C content very rapidly under inadequate dietary intake. However, a consumption of half a kiwifruit per day seems to be enough to saturate the muscle tissue’s vitamin C concentrations in non-smoking men (35). Vitamin C homeostasis is finely regulated by at least four mechanisms: intestinal absorption, transport to tissue, renal reuptake, and urine excretion, mainly regulated by a family of proteins named Sodium-Dependent Vitamin C Transporters (SVCT) (36). Considering the individual variability in healthy subjects, studies suggest that a daily intake of vitamin C from 100 to 400 mg ensures 100% of the bioavailability and blood saturation with a steady state of plasma concentration that reaches a maximum level of approximately 70–80 µmol/L (37, 38). Generally, if the intake of vitamin C exceeds 500 mg/day, a further increase in plasma concentration is inhibited and the bioavailability can decrease close to 30% when more than 1,000 mg of vitamin C is administered in a single bout (39). This occurs because when 500–1,000 mg of vitamin C is administered orally, the intestinal transporter (SVCT1) rapidly achieves its maximal saturation, while the urine excretion of the vitamin is progressively increased (38, 39). The measure of plasma vitamin C concentration may be considered, even though the circulant values cannot be used as a reliable marker of the body stores (about 5 g) (40). A plasma concentration value of vitamin C lower than 23 μmol/L reflects a depletion of the vitamin C pool (state of hypovitaminosis), while clinical symptoms of scurvy occur when plasma values are lower than 11 μmol/L (41).

### Recommended Dietary Allowances

To maintain adequate body stores, recommended dietary allowance (RDA) for vitamin C has been proposed over the years. The RDAs vary among countries: e.g., current recommendations in the United States and Canada is 90 mg/day for adult men and 75 mg/day for adult women (42), while in Italy the suggested intakes are 105 mg/day and 85 mg/day for adult men and women, respectively (43). This variation in RDAs can be explained by the different criteria used by various authorities to define the estimated average requirement (EAR) for vitamin C, including prevention of scurvy, immune cell support, maintenance of an adequate plasma vitamin C level, and optimizing health (44). Furthermore, RDAs vary among subjects as several factors can modify vitamin C requirements, including gender, age, smoking, pregnancy, and lactation (44).

Several authors and guidelines indicated that males need more vitamin C than females (45–51) probably due to the higher body weight and fat-free mass of men compared to women (52).

In children and adolescents the RDAs for vitamin C are generally derived from adult needs and adjusted in relation to their lower body mass (46, 47, 53) as reported by Carr and Lykkesfeldt (44). In Italy, for example, SINU recommends an intake of 45 mg for children from 4 to 6 years of age (45). Epidemiological studies indicate that a lower vitamin C status can be found in the elderly, suggesting that they require a higher intake compared to adults (54–56). However, currently only France has developed specific guidelines for subjects from 75 years of age, indicating a daily intake of 120 mg (57).

Vitamin C requirements are higher in women during pregnancy (44): the hemodilution due to the increase of blood volume and the active absorption of vitamin C by the fetus during its development lead to a reduction of the vitamin status (58). Even lactation increases the vitamin C requirement of women, to satisfy the vitamin needs of the infant normal growth. Most countries recommend an extra daily intake of 10–20 mg for pregnant women and an extra daily intake of 20–60 mg/day for women during lactation (44).

Smokers usually have lower plasma values than non-smokers, probably due to increased oxidative stress and higher turnover of vitamin C. In addition to this, a reduced vitamin C status in smokers is also due to the dietary intake of vitamin C, which is usually lower compared to non-smokers (59). Therefore, in order to compensate these conditions, authorities have recommended an additional intake of 20 to 80 mg/day of vitamin C for these subjects, setting the RDAs for smokers at 120–155 mg/day (44).

Some other factors have been recognized as reducing vitamin C status (60), even though they were not considered in general guidelines and the daily additional values of vitamin C potentially required are not currently available. These factors include: 1) passive exposure to tobacco smoking and environmental pollutants, which can enhance oxidative stress; 2) geographic influence, socioeconomic and cultural status, which may have an impact on production, selection and consumption of foods typically rich in vitamin C; 3) food preparation and cooking methods, which can reduce the content of vitamin C in foods since this vitamin is water-soluble and heat-labile.

The potential variability of the metabolism of vitamin C among various ethnic groups is practically unknown and this topic should certainly be further explored. Only one study reported that lower vitamin C concentrations were significantly associated with a higher leukocyte count in African Americans but not in Caucasians, suggesting hypothetical metabolic or pharmacokinetic differences among races (61).

## Vitamin C and Immune System Regulation

Besides an extensive range of biochemical pathways in which vitamin C is involved, it also participates in the response of the innate and adaptive immune system (1). The intracellular content of vitamin C in immune cells depends on the plasma availability. In healthy adults the content of vitamin C in leukocytes can be saturated with an intake of at least 100 mg of vitamin C per day, through foods, obtaining a concentration of about 3.5, 3, and 1.5 mmol/L, respectively, in lymphocytes, monocytes and neutrophils (39, 62–65). Leukocytes’ absorption of vitamin C from the blood is very efficient, through SVCT proteins (66), resulting in an intracellular content which is 50 to 100 times greater than the plasma concentration (67, 68). As an effective antioxidant, vitamin C contributes to protecting neutrophils from oxidative stress during the early stages of an immune response, when neutrophils activate phagocytosis and produce reactive oxygen species (ROS) to destroy antigens (69, 70). Once the phagocytic capacity is exhausted and neutrophils start to die, vitamin C seems to regulate the process in favor of apoptosis, through the activation of a caspase-dependent cascade, inhibiting the transition to necrosis, and resulting in a more efficient resolution of inflammation (71).

Vitamin C is also involved in the migration of phagocytes (neutrophils and macrophages) toward the infection sites in response to chemoattractants (72, 73). This is particularly important since an impaired neutrophilic chemotaxis has been observed in patients with severe infection (74–76). Furthermore in subjects with low blood concentrations of vitamin C (<50 µmol/L), a daily intake of 250 mg of vitamin C can result in a 20% increase of neutrophils’ migration capacity (6). Conversely, in individuals with a physiological blood concentration of vitamin C, neutrophils’ mobility cannot be enhanced, as demonstrated by Bozonet et al. (4), in which neutrophils isolated from healthy volunteers and incubated with a vitamin C solution (200 µmol/L) to artificially increase their content of ascorbate did not show a major chemotactic ability.

Similarly to neutrophils, vitamin C protects lymphocytes from oxidative damage (77) and has a pivotal role in the development and function of these cells, even though the exact mechanisms have not yet been clarified (3). In T lymphocytes, vitamin C stimulates differentiation and proliferation from precursors to mature T cells, in a dose-dependent way (78). Studies on the influence of vitamin C on subtypes of T cells are mainly related to the Th1/Th2 balance. Reports underline that vitamin C can induce a shift of immune responses from Th2 to Th1 (3), while only one study suggests that vitamin C can induce the Th17 polarization of naïve CD4+ cells in murine model, affecting epigenetic mechanism (79). At present, no studies exploring the effects of vitamin C on cytotoxic T cells are available (3). In B lymphocytes, vitamin C seems to affect the production of antibodies, despite conflicting evidence (80–87). Physiological levels of Vitamin C are also necessary for normal natural killer (NK) cell development and function (3). In vitamin C-deﬁcient mice, NK cytotoxic activity (NKCA) was lower than that in mice with normal levels of vitamin C (88), while supraphysiological levels of ascorbate do not further increase NKCA (89).

Vitamin C also regulates inflammatory response. In animal studies, vitamin C deficiency has been linked with higher circulating histamine levels, which can be rebalanced once vitamin C blood level has been normalized (90–92). Furthermore, vitamin C can reduce the production of pro-inflammatory leukocyte-derived cytokines (e.g., TNFα and IL-6), through the modulation of nuclear transcription factor kappa B (NFkB) (2, 93, 94) in at least two ways: 1) through its reduced form (ascorbate), by scavenging cellular ROS and inhibiting ROS-related signaling for the transcription of NFkB (95–98); 2) through its oxidized form (dehydroascorbate), produced as a consequence of quenching ROS, by directly inhibiting the activity of several kinases involved in the TNFα-mediated activation of NFkB (p38 mitogen-activated protein kinase, IkB kinase α and β) (99–101).

However, the effect of vitamin C on the balance of cytokine responses (pro- and anti-inflammatory) is very complex and seems to be dependent on cell type and/or inflammatory condition (1). Moreover, authors (102, 103) have recently suggested that vitamin C may interact with molecular pathways related to inflammatory stress and immune dysfunction during sepsis, involving particular mediators: Epidermal Growth Factor Receptor (EGFR), Mitogen-Activated Protein Kinase-1 (MAPK1), Proto-Oncogene c (JUN), C–C chemokine Receptor type 5 (CCR5), Mitogen Activated Protein Kinase 3 (MAPK3), Angiotensin II Receptor type 2 (AGTR2), and Signal Transducer and Activator of Transcription-3 (STAT3).

The effects of vitamin C on immune function may also be expressed through epigenetic regulations, although this topic is still poorly understood (104, 105). The epigenetic remodeling associated with immune cell activation and differentiation includes DNA and histone modification (106). Vitamin C plays an important role by increasing the activity of epigenetic enzymes, including ten-eleven translocation (TET) proteins and Jumonij-C domain-containing histone demethylases (JHDMs) (107). In fact, since TET and JHDMs belong to the Fe^2+^/α-ketoglutarate-dependent dioxygenases superfamily (105), vitamin C, as ascorbate, being able to donate electrons, acts as a cofactor for these enzymes, reducing Fe^3+^ to its catalytically active form (Fe^2+^) (63). TET proteins are involved in the demethylation of cytosine residues in DNA, while JHDMs regulate the methylation of lysine and arginine residues in histones, resulting in modifications of gene transcription (63, 108, 109) that are involved in the response of both the innate and the adaptive immune system (106, 110). The utility of these recently-discovered gene-regulatory functions of vitamin C for the assessment of dietary recommendations has not yet been elucidated and further research is needed to indicate the minimum dose at which vitamin C may have an effective impact on functional or clinical outcomes through epigenetic changes (44).

## Oral Supplementation of Vitamin C for the Prevention and Treatment of the Common Cold and Upper Respiratory Tract Infections

The common cold is one of the most widespread viral upper respiratory tract infections (URTI), characterized by coughing, tiredness, fever, sore throat, and muscle pain, which persist for a period ranging from a few days to not more than 3 weeks (111, 112). The term “common cold” refers to an unspecific syndrome caused by several viruses, although the rhinovirus is the most frequent pathogen involved, being found in 30% to 50% of sufferers (113). Despite symptomatology usually being very mild, the common cold is a major cause of absenteeism from work and school (114). The popular myth that a very high intake of vitamin C may lead to a lower susceptibility to respiratory tract infections originates from Linus Pauling’s theories published in the seventies. According to Pauling, a daily vitamin C intake of 1,000 mg can reduce the incidence of colds by about 45% and the optimal daily intake of vitamin C to live healthily and prevent disease should be at least 2.3 g (115, 116). The response of the US market to this pioneering point of view was immediate and the sales of vitamin C dietary supplements almost doubled over a couple of years (117). However, other clinical studies with similar aims failed to demonstrate its efficacy (118–121) and, in general, contemporary authors completely refuted Pauling’s ideas, mainly based on non-randomized controlled trials or incorrect application of animal background to humans (122, 123). Although a high daily dose of vitamin C does not seem to prevent viral infections in the general population, some categories of subjects with potentially higher risk of viral infection may require particular consideration. These subjects include individuals undergoing a daily heavy physical workload such as soldiers and athletes, who may develop an immune stress condition.

### General Population

If we exclude some results that reported only small or inconsistent effects attributable to vitamin C (124), after decades of investigations, the scientific community established that a high intake of vitamin C is useless in preventing the common cold (7–11), and therefore, a regular daily supplementation is not justified in the general population (12). Recent meta-analysis has reached similar conclusions regarding the incidence of infection, underlining, however, the possibility of reducing the duration of a cold. Gómez et al. (125), demonstrated that 8,472 subjects from eight randomized clinical trials (RCTs), showed very strong evidence that vitamin C intake above 80 mg/day does not prevent the common cold in healthy adults and children. Vorilhon et al. (126), analyzed eight RCTs and confirmed that vitamin C supplementation (dosage from 0.5 g to 2 g/day) is not effective, compared to placebo, in reducing the incidence of upper respiratory tract infections (URTI) in 3,135 children (from 3 months to 18 years of age), although the administration can reduce the duration of URTI by 14%, as previously highlighted by Rondanelli et al. (127). Positive results on the duration of colds was also suggested by the meta-analysis of Ran et al. (128) in which the combination of a small, long-term daily dose of vitamin C (no more than 1 g/day) to sustain immunity and a larger dose of vitamin C during the onset of the common cold (usually 3–4 g/day) was associated with the ability to relieve chest pain, fever, and chills, reducing the staying indoors duration, as well as the mean duration of disease.

### People Under Heavy Physical Stress

Some authors have reported a high incidence of respiratory infections in military training centers, probably also due to an overcrowding of individuals often coming from different geographical areas (129–131). More data are available on athletes, for whom daily high-intensity training and competitions have been associated with transient immune perturbations, inflammation conditions, and increased susceptibility to infections (132–134). Furthermore, compared to the general population, athletes have a higher exposure to pathogens, due to frequent travel and sports events (135, 136), which may potentially increase the risk of developing viral infections.

Data in literature referring to the effect of vitamin C supplementation on the prevention of the common cold in these subjects are interesting, despite being limited at present. As was well described by Hemilä and Chalker, (12) vitamin C supplementation may decrease the incidence of colds by about 50% in people under extreme physical stress. More recently, Kim et al. (137) carried out a large randomized, double-blind, placebo-controlled trial in 1,444 Korean soldiers, 695 of whom received vitamin C (6 g/day) for 30 days. They showed that the vitamin C group had a 0.80-fold lower risk of getting the common cold compared to the placebo group (n = 749).

The theoretical basis for the use of vitamin C in physically stressed subjects resides in the significant increase of ROS production due to intense exercise (138), with remarkable tissue damage/inflammatory response that may have harmful consequences on preventing URTI (139), possibly requiring a higher antioxidant intake compared to the general population (140). Despite this, it has recently been established that the administration of a high dose of antioxidants can negatively interfere with exercise-induced redox signaling and muscle adaptations (141–147) and the use of high doses of vitamin C to abolish ROS, especially over a long period, should be avoided (148, 149).

Even though the effects of isolated vitamin C on oxidative stress, inflammatory markers, muscle damage and immune response following exercise remain to be clarified in depth (146), a recent scientific society position stand (150), a recent review (140), and a meta-analysis (146) agree on recommending vitamin C supplementation (0.25–1.0 g/day) as an option to prevent URTI symptoms in athletes under heavy exertion and/or during periods of increased risk of infection (e.g., travel abroad) (140); athletes with low initial blood concentrations of vitamin C are the major candidates for supplementation (146, 149, 151, 152).

## Oral Supplementation of Vitamin C for the Prevention and Treatment of Pneumonia

Pneumonia is a lower respiratory tract infection characterized by a cough, difficulty in breathing, chest pain, fever, and lung inflammation (153). Pneumonia is the first cause of death by infection in the United States and the fifth most common cause of death overall (154, 155). *Streptococcus pneumoniae* and *Haemophilus influenzae* are recognized as the most common agents responsible for pneumonia (156) but other pathogens are also able to induce pneumonia, including viruses and fungi (153, 154).

Results obtained in rats and mice suggested that orally supplemented vitamin C may be useful in reducing susceptibility to viral pneumonia and potentially in reducing the development of ARDS (157, 158), which is recognized as the most severe form of acute respiratory infection (159). However, human findings on vitamin C supplementation and pneumonia remain scarce, with few dated observations mainly based on particular subjects and conditions (e.g., military people, developing countries) and not generalizable (5, 160). On this topic, the most recent meta-analysis including 2,774 participants from seven clinical studies, underlined that current evidence is insufficient to sustain the efficacy of vitamin C supplementation in preventing or treating pneumonia, due to the small number of trials and very low quality of the existing results (161). However, the meta-analysis of Padhani et al. (161) considered studies from different populations, including three studies on children, without subgroup analysis. Since the pharmacokinetics of vitamin C varies between subgroups and is not yet known in children, an analysis of data should have been done independently and, therefore, conclusions of this study may be questionable.

## Oral Supplementation of Vitamin C for the Prevention of COVID-19

COVID-19 is a new, worldwide recognized form of viral pneumonia, caused by SARS-CoV2 infection (162, 163). The symptomatology often begins within 2 weeks from contagion and mainly includes fever, fatigue, cough, and shortness of breath (164). Current knowledge suggests that while the majority of infected subjects (80%–90%) exhibit mild symptoms or can be asymptomatic, about 5% may develop pneumonia, ARDS and multi-organ dysfunction leading to death (165).

It is unquestionable that an optimal nutritional status effectively reduces inflammation and oxidative stress, improving the immune system regulation (166). However, no data are currently available on the regular use of high doses of oral vitamin C to reduce the risk of infection by SARS-CoV2 in a healthy general population (167–169) and further studies are needed to explore the role of vitamin C in prevention of COVID-19 (169). For heavily stressed subjects (athletes in particular), specific data are not currently reported regarding the incidence, prevalence, or natural history of disease related to COVID-19 (134), despite these subjects’ potentially high risk of exposure to this virus (136, 170). Furthermore, scientific opinions have not been expressed regarding the use of oral vitamin C to prevent SARS-CoV2 infection in extreme exercisers. However, a vitamin C supplementation may be effective for improving the health status of patients considered at high risk of viral infections (171).

## Oral Supplementation of Vitamin C for Patients With Metabolic Disorders, Cardiovascular Disease, and Frail Elderly Subjects: Potential Relationship With COVID-19

There are notably some factors that increase the risk of developing SARS-CoV2 infection and affect the severity of COVID-19 (172). People with pre-existing non-communicable diseases (NCDs) appear to be more susceptible to developing COVID-19 (173, 174). NCDs include obesity, diabetes mellitus, chronic lung diseases, cardiovascular diseases (CVD) and various other conditions which are characterized by systemic inflammation which impairs immune response and may exacerbate the cytokine storm related to COVID-19 (173, 174).

### Obese Subjects

Some studies have shown that obesity is associated to a more severe form of COVID-19 (175, 176), even in younger patients (age < 50) (177), and a BMI > 40 kg/m^2^ was identified as a one of the strongest risks of hospitalization due to SARS-CoV2 infection (178). These findings are worrying considering that obesity is a global phenomenon and in countries such as the U.S about 36% of population is obese (179). This association could be linked to inflammatory mechanisms, since authors suggest that, compared to individuals with a normal weight, obese subjects have a higher plasma concentration of C-reactive Protein (CRP), an inflammatory biomarker used to predict cardiovascular disease (180). Based on this, a vitamin C supplementation in these subjects may be useful in reducing inflammation, considering data that showed how a treatment of oral vitamin C (1,000 mg/day) for two months can significantly reduce plasma CRP in healthy, overweight, non-smokers with baseline CRP ≥ 1.0 mg/L (181). This finding is very interesting, taking into account that participants had an adequate dietary intake of vitamin C, with a baseline mean plasma level of 57.8 μmol/L, and it suggests that the RDAs for vitamin C in obese individuals may be underestimated, as was recently underlined by Rychter et al. (182, 183). Research is needed to understand whether by reducing the CRP with vitamin C it could be possible to influence the incidence and/or the progression of inflammation-mediated diseases associated with obesity, including infections and potentially COVID-19 (171).

### Diabetic Subjects

A recent meta-analysis including 33 studies (16,003 patients) confirmed that diabetics have a two-fold higher increase in mortality, as well as severity of COVID-19, compared to non-diabetic COVID-19 patients (184). Type 2 diabetes mellitus (T2DM) is the most common form of diabetes (185), characterized by chronic hyperglycemia, inflammation and oxidative stress (186). The inflammatory condition observable in diabetes may possibly be a mechanism that increases the susceptibility to COVID-19. Low plasma concentrations of vitamin C in people with T2DM was observed (187, 188), despite adequate vitamin C intake (189, 190). Two mechanisms could particularly explain lower vitamin C levels in these patients: 1) increased urinary excretion, especially in those with microalbuminuria (191); 2) higher depletion of vitamin C caused by an increase of oxidative stress (190, 192). An interesting study on the use of oral vitamin C in diabetic subjects was reported by Mason et al. (193). It showed an approximately two-fold enhanced SVCT2 expression in skeletal muscle after vitamin C supplementation (1,000 mg for 4 months) in people with T2DM, with an increase of muscle concentration of vitamin C and a decrease of muscle oxidative stress. However, given the small number of subjects investigated in this study (seven participants, six males and one female), larger studies are needed to confirm similar results. Findings from an RCT showed that vitamin C supplementation (1,000 mg/day for 8 weeks) significantly reduced CRP, IL-6, fasting blood glucose (FBG), and triglycerides (TG) in 64 obese, hypertensive and/or diabetic patients, with high levels of CRP ≥ 6 mg/L (194). In addition, meta-analytic data indicated that vitamin C supplementation for more than 30 days with a dosage ranging from 200 to 1,000 mg signiﬁcantly reduces FBG in patients with T2DM (195). Based on the above, vitamin C supplementation may represent a promising option to modulate inflammation and blood glucose in patients with hyperglycemia and elevated CRP, it could potentially be able to improve the health of these individuals and reduce the susceptibility to infections. Investigations are strongly encouraged to establish a possible correlation between an extra intake of vitamin C and a possible decrease of incidence, severity and mortality for COVID-19.

### Subjects With CVDs

CVDs (as well as hypertension) are the most common comorbidities among COVID-19 patients (174, 196, 197). Individuals with a CVD are five-fold more at risk of developing the critical stage of the disease, as indicated in a meta-analysis involving over 3,000 patients with COVID-19 (198). In this case, the main reason for a higher risk of SARS-Cov2 infection is related to the high angiotensin-converting enzyme 2 (ACE2) expression observed in these patients (199–201), since this enzyme is used by the virus to invade cells, promoting viral colonization (202). It is known that low plasma concentrations of vitamin C are predictive of heightened CVD risk (203–205), but the current literature provides little support for a widespread use of vitamin C supplements to reduce CVD risk or mortality (206), and available data are also controversial. Many cohort studies and RCTs have shown no relationship between vitamin C intake and CVD risk. However, in most RCTs the participants were not prescreened for a depleted status and this seriously limits the possibility of concluding on the results, as the potential impact of the vitamin C supplementation on the outcomes considered may vary from highly significant to negligible in relation to their vitamin C status at study start (207).

A few other studies have suggested moderate benefits, and some references have registered a slight increase in risk (206). A significant barrier to the comprehension of the relationship between vitamin C and CVDs is the lack of mechanistic studies in humans (206). At present, there are no recommendations for an additional daily dose of vitamin C in CVDs to potentially prevent diseases, including pulmonary infections and COVID-19.

### Frail Elderly Subjects

It is particularly important to consider elderly communities when trying to prevent COVID-19. The elderly are more vulnerable compared to the general population due to an increased risk of malnourishment and infections and a high prevalence of NCDs (208). Age itself is a risk factor for developing COVID-19 (209), due to a functional decline of the immune system (210, 211). Furthermore, malnourishment in these subjects is very frequent for several reasons (e.g., poor socioeconomic conditions, mental status, social status) (212) and nutritional deﬁciencies (including vitamin C) have been reported (213). Malnourishment can worsen an impaired immune system in the elderly, making them more susceptible to infections (214). In elderly hospitalized subjects (mean age 81 years) suffering from acute bronchitis or pneumonia, a mean plasma vitamin C level at baseline of 23 µmol/L was reported and a concentration of 11 µmol/L was found in one third of the patients (215). This is particularly important since a low vitamin C concentration (<17 µmol/L) in older people (aged 75–82 years) is considered a strong predictor of all-cause mortality (216). Notably, in Hunt’s study (215) administration of vitamin C (0.2 g/day) reduced the respiratory symptom score in the more severe patients. However, at present, it is not known whether a regular supplementation with vitamin C can protect these subjects from chronic inﬂammation NCDs-related and/or can prevent the onset of viral infections including COVID-19.

## Oral Supplementation and Side Effects

Vitamin C has an excellent safety profile, primarily due to its high water solubility and rapid clearance of excess levels by the kidneys (44, 217). Although it is not possible to establish a UL for vitamin C, values of 1,000–2,000 mg/day have been suggested as prudent limits by some countries, based on a potential risk of osmotic diarrhea and related gastrointestinal distress in some individuals at higher doses (44, 53).

Since vitamin C is partially converted to oxalate and excreted in the urine, high doses of vitamin C could be associated with calcium oxalate stone formation (218, 219). Ferraro et al. (220) studied 156,735 women and 40,536 men, who reported episodes of kidney stones during an average follow-up of 11.3–11.7 years. The authors signiﬁcantly correlated the total vitamin C intake with a higher risk of incident kidney stones in men, but not in women. However, it is important to outline that this study had limitations to be considered. The presence of confounding factors (e.g., comorbidities, dehydration, dietary intakes of oxalate-forming foods) were not taken into account during the follow-up, and the authors assessed vitamin C intake only through a questionnaire (without measuring blood levels) and with very long time intervals (every 4 years).

## Intravenous Administration of Vitamin C: A Potential Role in the Treatment of COVID-19?

While an extra dietary intake of vitamin C to counter pneumonia does not seem promising, several interesting data have emerged from the use of vitamin C through IA, providing an encouraging, but questioned, hypothesis on its potential pharmacological use for the treatment of pneumonia caused by SARS-CoV2 infection. In fact, as opposed to oral supplementation, in which the maximum peak plasma concentration that was achieved with a high-dose (3 g every 4 h) was 220 μmol/L (221), the IA of vitamin C, bypassing the limitations induced by intestinal transporters (SVCT1), may lead to a higher plasma level (e.g., up to 3,000 μmol/L at day 4 with 200 mg/kg/day, administered in 50 mg/kg/dose every 6 h).

Although the potential antisepsis therapeutic mechanism exerted by vitamin C has not yet been understood (103), besides the effects described in the previous paragraphs, the use of vitamin C in an infectious emergency may be justified for some reasons: 1) significant clinical evidence from pneumonia, critical illnesses and other acute infections suggests that plasma levels of vitamin C can rapidly drop off (e.g., <30 μmol/L) during the inflammatory response (2, 93, 94, 222–228) probably due to an increased consumption of vitamin C by leukocytes. Considering that intracellular ascorbate concentrations in mononuclear cells and in granulocytes are respectively 80 and 25 times greater than in plasma, an increased replacement and turnover of these cells during these medical conditions can contribute to a decrease of vitamin C in the blood (229); 2) a negative regulation of SVCT2 transporters induced by inflammatory cytokines, in particular IL-1β and TNFα (230); 3) the antioxidant defense system of the pulmonary epithelium involves enzymes and vitamin C (231) and according to Banerjee and Kaul, a sustained high dose of vitamin C available in respiratory secretion could exhibit an effective anti-viral activity (232). This last point, however, is still a hypothesis at present, since the level of vitamin C in the bronchoalveolar fluid is normally too low to achieve anti-viral activity and furthermore very little is known about the potential increase of vitamin C concentration in bronchial tissue and fluid secretion following a high-dose IA (36).

Therefore, considering the aspects mentioned above, the infusion of vitamin C has recently been suggested to treat COVID-19 in ICU hospitalized patients (13–18). Below, we summarize the most substantial evidence obtained in critical illness studies based on IA of vitamin C regarding the most relevant outcomes (inflammation, ventilation time, and mortality) which may relate to SARS-CoV2-induced ARDS.

### Effects of IA of Vitamin C on Inflammation Markers

**Figure 1:**
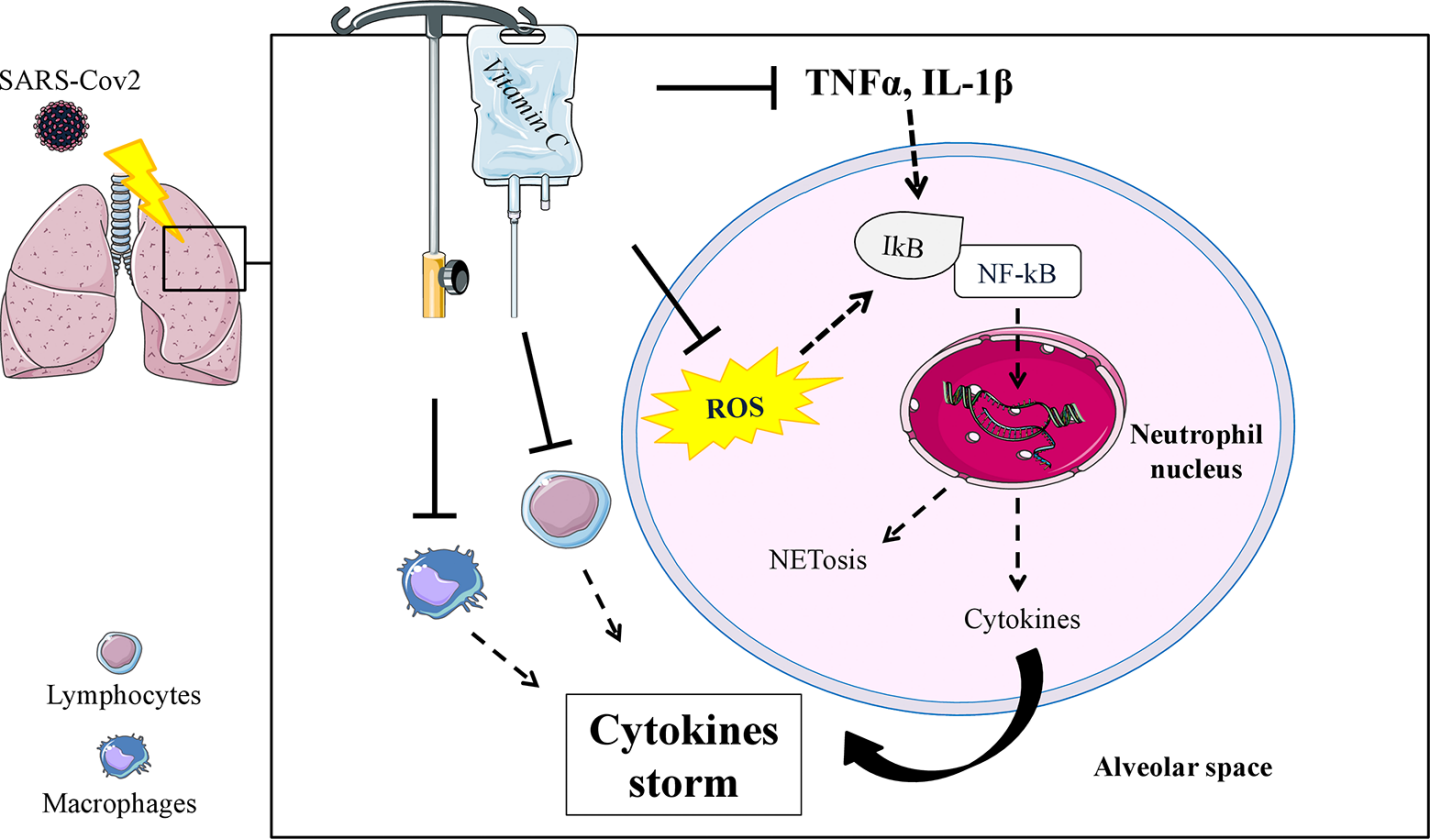
Schematic mechanism in which an IA of vitamin C could modulate specific functions of neutrophils (ROS and TNFα, IL-1β mediated), inhibiting pathways involved in the Neutrophil Extracellular Trap formation (NETosis) and reducing the uncontrollable inflammatory cytokine production in the alveolar space. Potential effects on reducing cytokine production have also been speculated in lymphocytes and macrophages. ROS, reactive oxygen species; NFkB, nuclear transcription factor kappa B; ┴, inhibition stimulus; dashed arrow, reduced effect or production.

In COVID-19 patients the inflammatory response is very dramatic and has been defined as a “cytokine storm”, associated with increased plasma concentration of IL-1β, IL-2, IL-6, IL-10, IFNγ, and TNF-α (233, 234), able to induce an acute lung injury (ALI) which results in ARDS and requires urgent ICU interventions (162). Physiopathology of ARDS involves alteration of pulmonary permeability, rapid lung leukocyte infiltration with a large increase of tissue oxidative stress, leading to respiratory failure and death, which in most cases is due to massive alveolar damage (24, 235). A promising background on the use of vitamin C in an experimental model of ALI was found (236–241), with positive evidence on rebalancing cytokine production and specific physiopathological mechanisms involving neutrophils (i.e., neutrophil extracellular traps) (4) which may contribute to organ damage and mortality in COVID-19 (233) (**Figure 1**). Two studies by Fowler et al. are currently available on IA of vitamin C in ICU hospitalized patients and inflammatory response, with mixed results (227, 242). In the first preliminary study (227), vitamin C showed a significant reduction in proinflammatory biomarkers (CRP and procalcitonin) over the first 96 h, without adverse effects registered during the infusion, but the number of ARDS-affected patients treated with vitamin C was too small (50 mg/kg/24 h, n = 8; 200 mg/kg/24 h, n = 8) to allow safe conclusions. In the second larger study (242), 167 patients with sepsis and ARDS were randomized to receive vitamin C (50 mg/kg every 6 h for 96 h) or placebo; no changes in CRP and thrombomodulin were detected, although the study was criticized for the choice of the inflammatory markers assessed (243).

### Effects of IA of Vitamin C on Intensive Care Ventilation Time

The above-mentioned study (242) also reported a lower duration time of mechanical ventilation support in the supplemented group, with a higher number of ventilator-free days in the vitamin C group than in the placebo group (mean values: 10.6 vs. 13.1 days, respectively). Ventilator-free days were defined as the number of extubated days, considering the time between ICU hospitalization and day 28. Another previous randomized controlled trial (RCT), including burn patients with severe respiratory dysfunction receiving a very high dose of vitamin C (66 mg/kg/h for 24 h), showed a significant decrease of the time of ventilation (mean values: 12.1 vs. 21.3 days, respectively) in those who received vitamin C infusion compared to the control group (85). The pulmonary benefit reported by these authors is probably due to the antioxidant, anti-inflammatory and microvascular action of the vitamin C (244).

Different positions on the topic derive from systematic evaluation of the literature, suffering because of the gross limitations of the available primary data. For example, the meta-analysis of Langlois et al. (245), failed to find any improvement on ventilation time. This work, however, included studies with vitamin C administrated through different routes (enteral or parenteral), most of which (9 out of a total of 11 studies) used antioxidant mixtures instead of vitamin C alone; Zhang and Jativa (244) analyzed the efficacy of IA of vitamin C on vasopressor sparing effects and the lower need for mechanical ventilation in critical illness, underlining several weaknesses of the available studies considered (four RCTs and one retrospective review), such as the paucity of the sample size, the heterogeneity of subjects enrolled, hospitalization setting, dosages and follow-up; recently, the meta-analysis from Hemilä and Chalker (246), including eight trials and 685 patients, with promising results on ventilation time, pointed out that the great variation in the reported effects of vitamin C may be linked to the non-homogeneous severity of the illness which mostly impacts the ventilation time required. From this point of view, vitamin C shortened ventilation time on average by 25% when the analysis was restricted to patients requiring mechanical support for more than 10 h.

### Effects of IA of Vitamin C on Mortality

Of the critical outcomes considered the potential effect of vitamin C on mortality rates appears to be the most controversial one, with RCTs studies that underline promising results which are not supported by recent meta-analysis. A significant reduction of 28-day mortality during ICU hospitalization was observed in a small group of patients with sepsis treated with IA of vitamin C (25 mg/kg every 6 h, for 72 h) compared to the control group (14.28% Vs. 64.28%, respectively) (247). More recently, findings from the CITRIS-ALI study (242) showed a reduced mortality at day 28 in the vitamin C group (29.8%) compared to the placebo group (46.3%). Conversely, according to the meta-analysis of Zhang and Jativa, although vitamin C IA seems to be linked to positive vasopressor effects, temporally reducing the need for mechanical ventilation, no positive effect in favor of overall mortality emerged (244), leading the authors to conclude that it does seem improbable that vitamin C, considered as a single agent, could be so dramatically decisive on the physiopathology of a critical illness as to influence the incidence of mortality (244). Similar conclusions were drawn by Wei et al. (248), who, by including recently published retrospective studies in their meta-analysis, suggest the lack of benefit on 28-day mortality, both in ICU and in-hospital patients with sepsis.

It is important to consider that the effect of vitamin C infusion seems to exert different results on mortality in relation to the type of critical patients involved, especially when administration is in association with other drugs. From this point of view, two retrospective studies showed that vitamin C (1.5 g every 6 h), in combination with hydrocortisone (50 mg every 6 h), and thiamine (200 mg every 12 h) may dramatically reduce mortality by 56% in ICU patients with severe pneumonia (249) and by 79% in severe sepsis (250), compared with patients who did not receive vitamin C and thiamine. Unlike these data, a recently published RCT (VITAMINS) showed no benefit from the combination of IA of vitamin C, hydrocortisone and thiamine in comparison to hydrocortisone alone among patients with septic shock (251). However, as underlined by Carr (252), since the VITAMINS trial did not include a monotherapy vitamin C subgroup, this trial does not provide any information as to whether IA of vitamin C offers some benefit to septic patients in the absence of corticosteroid administration, and further trials are needed in this direction.

Another critical issue that should be highlighted is the timing of treatment administration. On this topic, important results come from a retrospective cohort study of 208 patients in septic shock (253), which suggested that the ICU mortality ratio [based on the APACHE (Acute Physiology and Chronic Health Evaluation)–predicted ICU mortality] of patients who received vitamin C with thiamine and hydrocortisone, increased linearly with the delay in treatment from initial sepsis presentation. Indeed, the APACHE-adjusted ICU mortality was significantly reduced only in patients who received vitamin C, thiamine, and steroids within 6 h from sepsis presentation (253).

## Conclusion

Apart from some specific individuals and conditions (**Table 1**), the evidence described is insufficient to support the efficacy of a regular supplementation with vitamin C for the prevention or treatment of the common cold or pneumonia in the general population. Interesting data on the possible use of vitamin C to prevent infections regard special conditions (e.g., soldiers and athletes) and subjects with metabolic disorders, CVDs or frailty, in which the potential control of inflammation by a vitamin C supplementation could represent an effective aid in reducing the risk of infection, even for COVID-19. However, this last statement needs to be properly supported by future RCTs. Even though the IA of vitamin C could be an adjuvant therapy to quickly restore plasma levels of vitamin C during severe sepsis and ARDS in ICU hospitalized patients (254), its effects on inflammation response, ventilation time and mortality rates still remain uncertain and results from further RCTs, especially in COVID-19 patients, are urgently needed.

**Table 1:** Summary of research findings on the use of vitamin C in humans.

General population	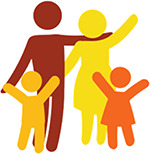	There are no recommendations that vitamin C supplementation impacts the incidence and duration of the common cold or pneumonia, and regular supplementation is not currently justified in the general population. Data on pneumonia are less clear in children, because the pharmacokinetics of vitamin C in this category is unknown at present. A supplementation of 0.2 g/day of vitamin C may be reasonable in subjects with low plasma vitamin C concentration.
Subjects with NCDs	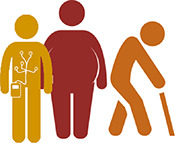	Subjects with noncommunicable diseases (NCDs) and frail, elderly subjects are recognized as being at high risk of viral infections. Vitamin C supplementation (0.2–1 g/day) can reduce inflammation and, although direct evidence is still lacking, it may decrease the susceptibility to infections and/or the severity of the disease. Vitamin C supplementation (0.2 g/day) may reduce the respiratory symptoms in elderly patients.
Heavy stressed subjects	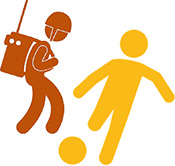	Vitamin C supplementation may decrease the incidence of colds in people under extreme physical stress. Vitamin C supplementation does not seem to improve physical performance in well-nourished athletes, and regular high-dose vitamin C supplementation may interfere with the exercise-induced redox signaling adaptation and should be avoided. Athletes should consider a vitamin C supplementation (0.25–1 g/day) to prevent URTI symptoms during limited periods of enhanced heavy stress (e.g., sports competitions).
Hospitalized patients	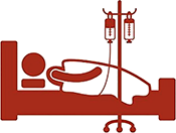	In critically ill patients, vitamin C deficiency is common, and IA of high doses has been used to normalize plasma vitamin C levels. To date, no recommendations are available as few data on inflammatory markers, mechanical ventilation time and mortality rates have been reported in these patients. Further controlled studies are encouraged, especially for COVID-19.

Although a significant increase in vitamin C sales was registered immediately after the global pandemic state of emergency was declared, at present, there is no evidence that vitamin C supplementation can protect people from the SARS-CoV2 infection (255). At the current state of knowledge, health care professionals have the responsibility to guarantee that patients have correct information regarding the lack of data supporting the efficacy of this supplement for the prevention and/or treatment of COVID-19 (167, 168).

## Author Contributions

All authors contributed to the article and approved the submitted version. GC and MN conceived the original idea of the manuscript and contributed equally to this work as main authors. GD’A revised the manuscript before submission.

## Conflict of Interest

The authors declare that the research was conducted in the absence of any commercial or financial relationships that could be construed as a potential conflict of interest.

## References

[1] Carr AC Maggini S Vitamin C and immune function. Nutrients (2017) 9:1211. 10.3390/nu9111211 PMC5707683 29099763

[2] Carr AC Rosengrave PC Bayer S Chambers S Mehrtens J Shaw GM Hypovitaminosis C and vitamin C deficiency in critically ill patients despite recommended enteral and parenteral intakes. Crit Care (2017) 21:200. 10.1186/s13054-017-1891-y 29228951 PMC5725835

[3] Van Gorkom GNY Klein Wolterink RGJ Van Elssen CHMJ Wieten L Germeraad WTV Bos GMJ Influence of Vitamin C on lymphocytes: An overview. Antioxidants (2018) 7:41. 10.3390/ANTIOX7030041 PMC5874527 29534432

[4] Bozonet SM Carr AC The role of physiological vitamin c concentrations on key functions of neutrophils isolated from healthy individuals. Nutrients (2019) 11:1363. 10.3390/nu11061363 PMC6627200 31212992

[5] Hemilä H Vitamin C and infections. Nutrients (2017) 52:222–3. 10.3390/nu9040339 PMC5409678 28353648

[6] Bozonet SM Carr AC Pullar JM Vissers MCM Enhanced human neutrophil vitamin C status, chemotaxis and oxidant generation following dietary supplementation with vitamin C-rich SunGold kiwifruit. Nutrients (2015) 7:2574–88. 10.3390/nu7042574 PMC4425162 25912037

[7] Allan GM Arroll B Prevention and treatment of the common cold: Making sense of the evidence. CMAJ (2014) 186:190–99. 10.1503/cmaj.121442 PMC3928210 24468694

[8] Douglas RM Hemilä H Vitamin C for preventing and treating the common cold. PloS Med (2005), CD000980. 10.1371/journal.pmed.0020168 PMC1160577 15971944

[9] Douglas RM Hemilä H Chalker E Treacy B Vitamin C for preventing and treating the common cold. Cochrane Database Syst Rev (2007) CD000980. 10.1002/14651858.CD000980.pub3 17636648

[10] Heimer KA Hart AM Martin LG Rubio-Wallace S Examining the evidence for the use of vitamin C in the prophylaxis and treatment of the common cold. J Am Acad Nurse Pract (2009) 21:295–300. 10.1111/j.1745-7599.2009.00409.x 19432914 PMC7166744

[11] Hemilä H Vitamin C supplementation and the common cold - Was Linus Pauling right or wrong? Int J Vitam Nutr Res (1997) 67:329–35. 9350474

[12] Hemilä H Chalker E Vitamin C for preventing and treating the common cold. Cochrane Database Syst Rev (2013) CD000980. 10.1002/14651858.CD000980.pub4 23440782 PMC8078152

[13] Boretti A Banik BK Intravenous vitamin C for reduction of cytokines storm in acute respiratory distress syndrome. PharmaNutrition (2020) 12:100190. 10.1016/j.phanu.2020.100190 32322486 PMC7172861

[14] Cheng RZ Can early and high intravenous dose of vitamin C prevent and treat coronavirus disease 2019 (COVID-19)? Med Drug Discovery (2020) 5:100028. 10.1016/j.medidd.2020.100028 PMC7167497 32328576

[15] Erol A High-dose intravenous vitamin C treatment for COVID-19. OSF Prepr (2020). 10.31219/osf.io/p7ex8

[16] HernándezAPapadakosPJTorresAGonzálezDAVivesMFerrandoC Two known therapies could be useful as adjuvant therapy in critical patients infected by COVID-19. Rev Esp Anestesiol Reanim (2020) 67:245–52. 10.1016/j.redar.2020.03.004 PMC715624232303365

[17] Matthay MA Aldrich JM Gotts JE Treatment for severe acute respiratory distress syndrome from COVID-19. Lancet Respir Med (2020) 8:433–34. 10.1016/S2213-2600(20)30127-2 PMC7118607 32203709

[18] Zhang L Liu Y Potential interventions for novel coronavirus in China: A systematic review. J Med Virol (2020) 92:479–90. 10.1002/jmv.25707 PMC7166986 32052466

[19] Worldometer COVID-19 Coronavirus Pandemic (2020). Available at: https://www.worldometers.info/coronavirus/.

[20] Yu F Du L Ojcius DM Pan C Jiang S Measures for diagnosing and treating infections by a novel coronavirus responsible for a pneumonia outbreak originating in Wuhan, China. Microbes Infect (2020) 22:74–9. 10.1016/j.micinf.2020.01.003 PMC7102556 32017984

[21] Alhazzani W Hylander Møller M Arabi YM Loeb M Ng Gong M Fan E Surviving Sepsis Campaign: Guidelines on the Management of Critically Ill Adults with Coronavirus Disease 2019 (COVID-19). Crit Care Med J (2020) 48:e440–69. 10.1097/CCM.0000000000004363 PMC7176264 32224769

[22] Singer M Deutschman CS Seymour C Shankar-Hari M Annane D Bauer M The third international consensus definitions for sepsis and septic shock (sepsis-3). JAMA (2016) 315:801–10. 10.1001/jama.2016.0287 PMC4968574 26903338

[23] Huang C Wang Y Li X Ren L Zhao J Hu Y Clinical features of patients infected with 2019 novel coronavirus in Wuhan, China. Lancet (2020) 395:497–506. 10.1016/S0140-6736(20)30183-5 31986264 PMC7159299

[24] The ARDS Definition Task Force* Acute Respiratory Distress Syndrome. The Berlin Definition of ARDS. JAMA (2012) 307:2526–33. 10.1001/jama.2012.5669 22797452

[25] Sun P Qie S Liu Z Ren J Li K Xi J Clinical characteristics of 50 466 hospitalized patients with 2019-nCoV infection. J Med Virol (2020) 92:612–17. 10.1002/jmv.25735 PMC7228255 32108351

[26] Available at: https://clinicaltrials.gov/.

[27] Linster CL Van Schaftingen E Vitamin C: Biosynthesis, recycling and degradation in mammals. FEBS J (2007) 274:1–22. 10.1111/j.1742-4658.2006.05607.x 17222174

[28] Frikke-Schmidt H Tveden-Nyborg P Lykkesfeldt J L-dehydroascorbic acid can substitute l-ascorbic acid as dietary vitamin C source in guinea pigs. Redox Biol (2016) 7:8–13. 10.1016/j.redox.2015.11.003 26609560 PMC4683385

[29] Michels AJ Hagen TM Frei B Human Genetic Variation Influences Vitamin C Homeostasis by Altering Vitamin C Transport and Antioxidant Enzyme Function. Annu Rev Nutr (2013) 33:45–70. 10.1146/annurev-nutr-071812-161246 23642198 PMC4357493

[30] Hasselholt S Tveden-Nyborg P Lykkesfeldt J Distribution of vitamin C is tissue specific with early saturation of the brain and adrenal glands following differential oral dose regimens in guinea pigs. Br J Nutr (2015) 113:1539–49. 10.1017/S0007114515000690 25865869

[31] Kim H Bae S Yu Y Kim Y Kim H-R Hwang Y The Analysis of Vitamin C Concentration in Organs of Gulo -/- Mice Upon Vitamin C Withdrawal. Immune Netw (2012) 12:18–26. 10.4110/in.2012.12.1.18 22536166 PMC3329599

[32] Harrison FE Green RJ Dawes SM May JM Vitamin C distribution and retention in the mouse brain. Brain Res (2010) 1348:181–86. 10.1016/j.brainres.2010.05.090 PMC2912448 20570663

[33] Toutain PL Béchu D Hidiroglou M Ascorbic acid disposition kinetics in the plasma and tissues of calves. Am J Physiol - Regul Integr Comp Physiol (1997) 273:R1587–97. 10.1152/ajpregu.1997.273.5.r1585 9374798

[34] Omaye St Schaus Ee Kutnink Ma Hawkes Wc. Measurement of Vitamin C in Blood Components by High-Performance Liquid Chromatography: Implication in Assessing Vitamin C Status. Ann N Y Acad Sci (1987) 498:389–401. 10.1111/j.1749-6632.1987.tb23776.x 3304068

[35] Carr AC Bozonet SM Pullar JM Simcock JW Vissers MCM Human skeletal muscle ascorbate is highly responsive to changes in vitamin C intake and plasma concentrations. Am J Clin Nutr (2013) 97:800–7. 10.3945/ajcn.112.053207 PMC3607654 23446899

[36] Lykkesfeldt J Tveden-Nyborg P The pharmacokinetics of vitamin C. Nutrients (2019) 11:2412. 10.3390/nu11102412 PMC6835439 31601028

[37] Frei B Birlouez-Aragon I Lykkesfeldt J Authors’ perspective: What is the optimum intake of vitamin C in humans? Crit Rev Food Sci Nutr (2012) 52:815–29. 10.1080/10408398.2011.649149 22698272

[38] Levine M Padayatty SJ Espey MG Vitamin C: A Concentration-Function Approach Yields Pharmacology and Therapeutic Discoveries. Adv Nutr (2011) 2:78–88. 10.3945/an.110.000109 22332036 PMC3065766

[39] Levine M Conry-Cantilena C Wang Y Welch RW Washko PW Dhariwal KR Vitamin C pharmacokinetics in healthy volunteers: Evidence for a recommended dietary allowance. Proc Natl Acad Sci U S A (1996) 93:3704–9. 10.1073/pnas.93.8.3704 PMC39676 8623000

[40] Nygaard G On a Novel, Simplified Model Framework Describing Ascorbic Acid Concentration Dynamics. In: 41st Annual International Conference of the IEEE Engineering in Medicine and Biology Society, EMBS (2019). p. 2880–6. 10.1109/EMBC.2019.8857675 31946493

[41] Lykkesfeldt J Poulsen HE Is vitamin C supplementation beneficial? Lessons learned from randomised controlled trials. Br J Nutr (2010) 103:1251–9. 10.1017/S0007114509993229 20003627

[42] Bechthold A New reference values for Vitamin C intake. Ann Nutr Metab (2015) 67:13–20. 10.1159/000434757 26227083

[43] VITAMINE Assunzione raccomandata per la popolazione (PRI) e assunzione adeguata (AI). sinu.it. Available at: https://sinu.it/2019/07/09/assunzione-raccomandata-per-la-popolazione-pri-e-assunzione-adeguata-ai/ (Accessed June 11, 2020).

[44] Carr AC Lykkesfeldt J Discrepancies in global vitamin C recommendations: a review of RDA criteria and underlying health perspectives. Crit Rev Food Sci Nutr (2020) 1–14. 10.1080/10408398.2020.1744513 32223303

[45] Italian Society of Human Nutrition (SINU) LARN Levels of reference intake for nutrients and energy for the Italian population. (2014). Available at: https://sinu.it/tabelle-larn-2014/.

[46] EFSA Panel on Dietetic Products, Nutrition and Allergies (NDA) Scientific Opinion on Dietary Reference Values for vitamin C. EFSA J (2013) 11:3418. 10.2903/j.efsa.2013.3418

[47] German Nutrition Society New Reference Values for Vitamin C Intake. Ann Nutr Metab (2015) 67:13–20. 10.1159/000434757 26227083

[48] Institute of Medicine (US) Panel on Dietary Antioxidants and Related Compounds Dietary Reference Intakes for Vitamin C, Vitamin E, Selenium, and Carotenoids. Washington (DC): National Academies Press (US) (2000). 10.17226/9810 25077263

[49] Galan P Viteri FE Bertrais S Czernichow S Faure H Arnaud J Serum concentrations of β-carotene, vitamins C and E, zinc and selenium are influenced by sex, age, diet, smoking status, alcohol consumption and corpulence in a general French adult population. Eur J Clin Nutr (2005) 59:1181–90. 10.1038/sj.ejcn.1602230 16034362

[50] Canoy D Wareham N Welch A Bingham S Luben R Day N Plasma ascorbic acid concentrations and fat distribution in 19 068 British men and women in the European Prospective Investigation into Cancer and Nutrition Norfolk cohort study. Am J Clin Nutr (2005) 82:1203–09. 10.1093/ajcn/82.6.1203 16332652

[51] Hampl JS Taylor CA Johnston CS Vitamin C Deficiency and Depletion in the United States: The Third National Health and Nutrition Examination Survey, 1988 to 1994. Am J Public Health (2004) 94:870–5. 10.2105/AJPH.94.5.870 PMC1448351 15117714

[52] Jungert A Neuhäuser-Berthold M The lower vitamin C plasma concentrations in elderly men compared with elderly women can partly be attributed to a volumetric dilution effect due to differences in fat-free mass. Br J Nutr (2015) 113:859–64. 10.1017/S0007114515000240 25735881

[53] National Health and Medical Research Council Nutrient Reference Values for Australia and New Zealand: Executive Summary. (2006).

[54] Faure H Preziosi P Roussel AM Bertrais S Galan P Hercberg S Factors influencing blood concentration of retinol, α-tocopherol, vitamin C, and β-carotene in the French participants of the SU.VI.MAX trial. Eur J Clin Nutr (2006) 60:706–17. 10.1038/sj.ejcn.1602372 16391586

[55] Birlouez-Aragon I Delcourt C Tessier F Papoz L Associations of age, smoking habits and diabetes with plasma vitamin C of elderly of the POLA study. Int J Vitam Nutr Res (2001) 71:53–9. 10.1024/0300-9831.71.1.53 11276923

[56] Ravindran RD Vashist P Gupta SK Young IS Maraini G Camparini M Prevalence and risk factors for vitamin C deficiency in North and South India: A two centre population based study in people aged 60 years and over. PloS One (2011) 6:e28588. 10.1371/journal.pone.0028588 22163038 PMC3232233

[57] Martin A The “apports nutritionnels conseillés (ANC)” for the French population. Reprod Nutr Dev (2001) 41:119–28. 10.1051/rnd:2001100 11434516

[58] Juhl B Lauszus FF Lykkesfeldt J Is diabetes associated with lower vitamin C status in pregnant women? A prospective study. Int J Vitam Nutr Res (2016) 86:184–89. 10.1024/0300-9831/a000407 28206812

[59] Lykkesfeldt J Priemé H Loft S Poulsen HE Effect of smoking cessation on plasma ascorbic acid concentration. Br Med J (1996) 313:91. 10.1136/bmj.313.7049.91 8688760 PMC2351485

[60] Carr AC Rowe S Factors affecting vitamin c status and prevalence of deficiency: A global health perspective. Nutrients (2020) 12:1963. 10.3390/nu12071963 PMC7400679 32630245

[61] Suarez EC Schramm-Sapyta NL Race differences in the relation of vitamins A, C, E, and β-carotene to metabolic and inflammatory biomarkers. Nutr Res (2014) 34:1–10. 10.1016/j.nutres.2013.10.001 24418240 PMC3894570

[62] Levine M Wang Y Padayatty SJ Morrow J A new recommended dietary allowance of vitamin C for healthy young women. Proc Natl Acad Sci U S A (2001) 98:9842–6. 10.1073/pnas.171318198 PMC55540 11504949

[63] Camarena V Wang G The epigenetic role of vitamin C in health and disease. Cell Mol Life Sci (2016) 73:1645–58. 10.1007/s00018-016-2145-x PMC4805483 26846695

[64] Mangge H Antioxidants, inflammation and cardiovascular disease. World J Cardiol (2014) 6:462–77. 10.4330/wjc.v6.i6.462 PMC4072837 24976919

[65] Ginter E Simko V Panakova V Antioxidants in health and disease. Bratislava Med J (2014) 115:603–6. 10.4149/BLL_2014_116 25573724

[66] Hong JM Kim JH Kang JS Lee WJ Hwang Y Vitamin C is taken up by human T cells via sodium-dependent vitamin C transporter 2 (SVCT2) and exerts inhibitory effects on the activation of these cells in vitro. Anat Cell Biol (2016) 49:88–98. 10.5115/acb.2016.49.2.88 27382510 PMC4927435

[67] Bergsten P Amitai G Kehrl J Dhariwal KR Klein HG Levine M Millimolar concentrations of ascorbic acid in purified human mononuclear leukocytes. Depletion and reaccumulation. J Biol Chem (1990) 265:2584–87. 2303417

[68] Evans RM Currie L Campbell A The distribution of ascorbic acid between various cellular components of blood, in normal individuals, and its relation to the plasma concentration. Br J Nutr (1982). 10.1079/bjn19820059 7082619

[69] Winterbourn CC Vissers MCM Changes in ascorbate levels on stimulation of human neutrophils. BBA - Mol Cell Res (1983) 763:175–9. 10.1016/0167-4889(83)90041-1 6615889

[70] Oberritter H Glatthaar B Moser U Schmidt KH Effect of functional stimulation on ascorbate content in phagocytes under physiological and pathological conditions. Int Arch Allergy Appl Immunol (1986) 81:46–50. 10.1159/000234106 3744577

[71] Vissers MCM Wilkie RP Ascorbate deficiency results in impaired neutrophil apoptosis and clearance and is associated with up-regulation of hypoxia-inducible factor 1α. J Leukoc Biol (2007) 8:1236–44. 10.1189/jlb.0806541 29350811

[72] Goldschmidt MC Reduced bactericidal activity in neutrophils from scorbutic animals and the effect of ascorbic acid on these target bacteria in vivo and in vitro. Am J Clin Nutr (1991) 54:1214S–20S. 10.1093/ajcn/54.6.1214s 1962573

[73] Goldschmidt MC Masin WJ Brown LR Wyde PR The effect of ascorbic acid deficiency on leukocyte phagocytosis and killing of Actinomyces viscosus. Int J Vitam Nutr Res (1988) 58:326–34. 2461911

[74] Demaret J Venet F Friggeri A Cazalis M-A Plassais J Jallades L Marked alterations of neutrophil functions during sepsis-induced immunosuppression. J Leukoc Biol (2015) 98:1081–90. 10.1189/jlb.4a0415-168rr 26224052

[75] Arraes SMA Freitas MS Da Silva SV De Paula Neto HA Alves-Filho JC Martins MA Impaired neutrophil chemotaxis in sepsis associates with GRK expression and inhibition of actin assembly and tyrosine phosphorylation. Blood (2006) 108:2906–13. 10.1182/blood-2006-05-024638 16849637

[76] Chishti AD Shenton BK Kirby JA Baudouin SV Neutrophil chemotaxis and receptor expression in clinical septic shock. Intensive Care Med (2004) 30:605–11. 10.1007/s00134-004-2175-y 14991094

[77] Lenton KJ Therriault H Fülöp T Payette H Wagner JR Glutathione and ascorbate are negatively correlated with oxidative DNA damage in human lymphocytes. Carcinogenesis (1999) 20:607–13. 10.1093/carcin/20.4.607 10223188

[78] Huijskens MJAJ Walczak M Koller N Briedé JJ Senden-Gijsbers BLMG Schnijderberg MC Technical Advance: Ascorbic acid induces development of double-positive T cells from human hematopoietic stem cells in the absence of stromal cells. J Leukoc Biol (2014) 96:1165–75. 10.1189/jlb.1ta0214-121rr 25157026

[79] Song MH Nair VS Oh KI Vitamin C enhances the expression of IL17 in a Jmjd2-dependent manner. BMB Rep (2017) 50:49–54. 10.5483/BMBRep.2017.50.1.193 27931518 PMC5319665

[80] Vallance S Relationships between ascorbic acid and serum proteins of the immune system. Br Med J (1977) 2:437–8. 10.1136/bmj.2.6084.437 PMC1631223 890328

[81] Anderson R Oosthuizen R Maritz R Theron A Van Rensburg AJ The effects of increasing weekly doses of ascorbate on certain cellular and humoral immune functions in normal volunteers. Am J Clin Nutr (1980) 33:71–6. 10.1093/ajcn/33.1.71 7355784

[82] Prinz W Bloch J Gilich G Mitchell G A systematic study of the effect of vitamin C supplementation on the humoral immune response in ascorbate-dependent mammals. I. The antibody response to sheep red blood cells (a T-dependent antigen) in guinea pigs. Int J Vitam Nutr Res (1980) 50:294–300. 7429758

[83] Feigen GA Smith BH Dix CE Flynn CJ Peterson NS Rosenberg LT Enhancement of antibody production and protection against systemic anaphylaxis by large doses of vitamin C. Res Commun Chem Pathol Pharmacol (1982) 38:313–33. 10.1016/s0022-5347(17)52586-0 7163630

[84] Kennes B Dumont I Brohee D Hubert C Neve P Effect of vitamin С supplements on cell-mediated immunity in old people. Gerontology (1983) 29:305–10. 10.1159/000213131 6604680

[85] Tanaka H Matsuda T Miyagantani Y Yukioka T Matsuda H Shimazaki S Reduction of resuscitation fluid volumes in severely burned patients using ascorbic acid administration: A randomized, prospective study. Arch Surg (2000) 135:326–31. 10.1001/archsurg.135.3.326 10722036

[86] Albers R Bol M Bleumink R Willems AA Pieters RHH Effects of supplementation with vitamins A, C, and E, selenium, and zinc on immune function in a murine sensitization model. Nutrition (2003) 19:940–6. 10.1016/S0899-9007(03)00178-3 14624943

[87] Hesta M Ottermans C Krammer-Lukas S Zentek J Hellweg P Buyse J The effect of vitamin C supplementation in healthy dogs on antioxidative capacity and immune parameters. J Anim Physiol Anim Nutr (Berl) (2009) 93:26–34. 10.1111/j.1439-0396.2007.00774.x 19386005

[88] Kim JE Cho HS Yang HS Jung DJ Hong SW Hung CF Depletion of ascorbic acid impairs NK cell activity against ovarian cancer in a mouse model. Immunobiology (2012) 217:873–81. 10.1016/j.imbio.2011.12.010 22306178

[89] Heuser G Vojdani A Enhancement of natural killer cell activity and T and B cell function by buffered vitamin C in patients exposed to toxic chemicals: The role of protein kinase - C. Immunopharmacol Immunotoxicol (1997) 19:291–312. 10.3109/08923979709046977 9248859

[90] Chatterjee IB Gupta SD Majumder AK Nandi BK Subramanian N Effect of ascorbic acid on histamine metabolism in scorbutic guinea-pigs. J Physiol (1975) 25:271–9. 10.1113/jphysiol.1975.sp011091 PMC1348426 52707

[91] Nandi BK Subramanian N Majumder AK Chatterjee IB Effect of ascorbic acid on detoxification of histamine under stress conditions. Biochem Pharmacol (1974) 23:643–7. 10.1016/0006-2952(74)90629-7 4132605

[92] Johnston CS Solomon RE Corte C Vitamin c depletion is associated with alterations in blood histamine and plasma free carnitine in adults. J Am Coll Nutr (1996) 15:586–91. 10.1080/07315724.1996.10718634 8951736

[93] Bonham MJD Abu-Zidan FM Simovic MO Sluis KB Wilkinson A Winterbourn CC Early ascorbic acid depletion is related to the severity of acute pancreatitis. Br J Surg (1999) 86:1296–301. 10.1046/j.1365-2168.1999.01182.x 10540137

[94] Bakaev VV Duntau AP Ascorbic acid in blood serum of patients with pulmonary tuberculosis and pneumonia. Int J Tuberc Lung Dis (2004) 8:263–6. 15139458

[95] Peng Y Kwok KHH Yang PH Ng SSM Liu J Wong OG Ascorbic acid inhibits ROS production, NF-κB activation and prevents ethanol-induced growth retardation and microencephaly. Neuropharmacology (2005) 48:426–34. 10.1016/j.neuropharm.2004.10.018 15721175

[96] Maiuolo J Maretta A Gliozzi M Musolino V Carresi C Bosco F Ethanol-induced cardiomyocyte toxicity implicit autophagy and NFkB transcription factor. Pharmacol Res (2018) 133:141–50. 10.1016/j.phrs.2018.04.004 29679641

[97] Moniruzzaman M Ghosal I Das D Chakraborty SB Melatonin ameliorates H2O2-induced oxidative stress through modulation of Erk/Akt/ NFkB pathway. Biol Res (2018) 51:17. 10.1186/s40659-018-0168-5 29891016 PMC5996524

[98] Thoma A Lightfoot AP Nf-kb and inflammatory cytokine signalling: Role in skeletal muscle atrophy. In: Xiao J, editor. Advances in Experimental Medicine and Biology, vol. 1088 Singapore: Springer (2018). p. 267–79. 10.1007/978-981-13-1435-3_12 30390256

[99] Bowie AG O’Neill LAJ Vitamin C Inhibits NF-κB Activation by TNF Via the Activation of p38 Mitogen-Activated Protein Kinase. J Immunol (2000) 165:7180–8. 10.4049/jimmunol.165.12.7180 11120850

[100] Cárcamo JM Pedraza A Bórquez-Ojeda O Golde DW Vitamin C suppresses TNFα-induced NFκB activation by inhibiting IκBα phosphorylation. Biochemistry (2002) 41:12995–3002. 10.1021/bi0263210 12390026

[101] Cárcamo JM Pedraza A Bórquez-Ojeda O Zhang B Sanchez R Golde DW Vitamin C Is a Kinase Inhibitor: Dehydroascorbic Acid Inhibits IκBα Kinase β. Mol Cell Biol (2004) 24:6645–52. 10.1128/mcb.24.15.6645-6652.2004 PMC444845 15254232

[102] Li R Guo C Li Y Liang X Yang L Huang W Therapeutic target and molecular mechanism of vitamin C-treated pneumonia: a systematic study of network pharmacology. Food Funct (2020) 11:4765–72. 10.1039/d0fo00421a 32420559

[103] Li R Guo C Li Y Qin Z Huang W Therapeutic targets and signaling mechanisms of vitamin C activity against sepsis: a bioinformatics study. Brief Bioinform (2020) bbaa079. 10.1093/bib/bbaa079 32393985 PMC7454291

[104] Kuiper C Vissers MCM Ascorbate as a cofactor for Fe-and 2-oxoglutarate dependent dioxygenases: Physiological activity in tumour growth and progression. Front Oncol (2014) 4:359. 10.3389/fonc.2014.00359 25540771 PMC4261134

[105] Young JI Züchner S Wang G Regulation of the Epigenome by Vitamin C. Annu Rev Nutr (2015) 35:545–64. 10.1146/annurev-nutr-071714-034228 PMC4506708 25974700

[106] Lio CWJ Rao A TET enzymes and 5hMC in adaptive and innate immune systems. Front Immunol (2019) 10:210. 10.3389/fimmu.2019.00210 30809228 PMC6379312

[107] Lee Chong T Ahearn EL Cimmino L Reprogramming the Epigenome With Vitamin C. Front Cell Dev Biol (2019) 7:128. 10.3389/fcell.2019.00128 31380368 PMC6646595

[108] Blaschke K Ebata KT Karimi MM Zepeda-Martínez JA Goyal P Mahapatra S Vitamin C induces Tet-dependent DNA demethylation and a blastocyst-like state in ES cells. Nature (2013) 500:222–6. 10.1038/nature12362 PMC3893718 23812591

[109] Minor EA Court BL Young JI Wang G Ascorbate induces ten-eleven translocation (Tet) methylcytosine dioxygenase-mediated generation of 5-hydroxymethylcytosine. J Biol Chem (2013) 288:13669–74. 10.1074/jbc.C113.464800 PMC3650403 23548903

[110] Ang A Pullar JM Currie MJ Vissers MCM Vitamin C and immune cell function in inflammation and cancer. Biochem Soc Trans (2018) 46:1147–59. 10.1042/BST20180169 PMC6195639 30301842

[111] Eccles R Understanding the symptoms of the common cold and influenza. Lancet Infect Dis (2005) 5:718–25. 10.1016/S1473-3099(05)70270-X PMC7185637 16253889

[112] Arroll B Common cold. BMJ Clin Evid (2011) 2011:1510. 10.5124/jkma.1998.41.11.1188 PMC3275147 21406124

[113] Heikkinen T Järvinen A The common cold. Lancet (2003) 361:51–9. 10.1016/S0140-6736(03)12162-9 PMC7112468 12517470

[114] Dicpinigaitis PV Eccles R Blaiss MS Wingertzahn MA Impact of cough and common cold on productivity, absenteeism, and daily life in the United States: ACHOO Survey. Curr Med Res Opin (2015) 31:1519–25. 10.1185/03007995.2015.1062355 26073933

[115] Pauling L Evolution and the need for ascorbic acid. Proc Natl Acad Sci U S A (1970) 67:1643–8. 10.1073/pnas.67.4.1643 PMC283405 5275366

[116] Pauling L The significance of the evidence about ascorbic acid and the common cold. Proc Natl Acad Sci U S A (1971) 68:2678–81. 10.1073/pnas.68.11.2678 PMC389499 4941984

[117] Duerbeck NB Dowling DD Duerbeck JM Vitamin C: Promises not kept. Obstet Gynecol Surv (2016) 71:187–93. 10.1097/OGX.0000000000000289 26987583

[118] Anderson TW Reid DB Beaton GH Vitamin C and the common cold: a double-blind trial. Can Med Assoc J (1972) 107:503–8. PMC1940935 5057006

[119] Karlowski TR Chalmers TC Frenkel LD Kapikian AZ Lewis TL Lynch JM Ascorbic Acid for the Common Cold: A Prophylactic and Therapeutic Trial. JAMA J Am Med Assoc (1975) 231:1038–42. 10.1001/jama.1975.03240220018013 163386

[120] Chalmers TC Effects of ascorbic acid on the common cold. An evaluation of the evidence. Am J Med (1975) 58:532–6. 10.1016/0002-9343(75)90127-8 1092164

[121] Dykes MHM Meier P Ascorbic Acid and the Common Cold: Evaluation of Its Efficacy and Toxicity. JAMA J Am Med Assoc (1975) 231:1073–9. 10.1001/jama.1975.03240220051025 1089817

[122] Bourne GH Vitamin C and Immunity. Br J Nutr (1949) 2:341–7. 10.1079/bjn19480063 18145790

[123] Stone I Hypoascorbemia, the genetic disease causing the human requirement for exogenous ascorbic acid. Perspect Biol Med (1966) 10:133–4. 10.1353/pbm.1966.0037 6002772

[124] Elwood PC Hughes SJ Leger St AS A randomized controlled trial of the therapeutic effect of vitamin C in the common cold. Practitioner (1977) 218:133–7. 319446

[125] Gómez E Quidel S Bravo Soto G Ortigoza Á. Does vitamin C prevent the common cold? Medwave (2018) 18:e7235. 10.5867/medwave.2018.04.7236 30113569

[126] Vorilhon P Arpajou B Vaillant Roussel H Merlin É Pereira B Cabaillot A Efficacy of vitamin C for the prevention and treatment of upper respiratory tract infection. A meta-analysis in children. Eur J Clin Pharmacol (2019) 75:303–11. 10.1007/s00228-018-2601-7 30465062

[127] Rondanelli M Miccono A Lamburghini S Avanzato I Riva A Allegrini P Self-Care for Common Colds: The Pivotal Role of Vitamin D, Vitamin C, Zinc, and Echinacea in Three Main Immune Interactive Clusters (Physical Barriers, Innate and Adaptive Immunity) Involved during an Episode of Common Colds - Practical Advice on Dosages. Evidence-Based Complement Altern Med (2018) 2018:5813095. 10.1155/2018/5813095 PMC5949172 29853961

[128] Ran L Zhao W Wang J Wang H Zhao Y Tseng Y Extra Dose of Vitamin C Based on a Daily Supplementation Shortens the Common Cold: A Meta-Analysis of 9 Randomized Controlled Trials. BioMed Res Int (2018) 2018:1837634. 10.1155/2018/1837634 30069463 PMC6057395

[129] Hemilä H Vitamin C Supplementation and Respiratory Infections: a Systematic Review. Mil Med (2004) 169:920–5. 10.7205/milmed.169.11.920 15605943

[130] Pazzaglia G Pasternack M Recent Trends of Pneumonia Morbidity in US Naval Personnel. Mil Med (1983) 148:647–51. 10.1093/milmed/148.8.647 6415517

[131] Kleemola M Saikku P Visakorpi R Wang SP Grayston JT Kleemola M Epidemics of pneumonia caused by twar, a new chlamydia organism, in military trainees in finland. J Infect Dis (1988) 157:230–6. 10.1093/infdis/157.2.230 3335808

[132] Scheffer D da L Latini A Exercise-induced immune system response: Anti-inflammatory status on peripheral and central organs. Biochim Biophys Acta - Mol Basis Dis (2020) 1866:165823. 10.1016/j.bbadis.2020.165823 32360589 PMC7188661

[133] Simpson RJ Campbell JP Gleeson M Krüger K Nieman DC Pyne DB Can exercise affect immune function to increase susceptibility to infection? Exerc Immunol Rev (2020) 26:8–22. 32139352

[134] Hull JH Loosemore M Schwellnus M Respiratory health in athletes: facing the COVID-19 challenge. Lancet Respir Med (2020) 8:557–8. 10.1016/S2213-2600(20)30175-2 PMC7194966 32277869

[135] Schwellnus MP Derman WE Jordaan E Page T Lambert MI Readhead C Elite athletes travelling to international destinations <5 time zone differences from their home country have a 2–3-fold increased risk of illness. Br J Sports Med (2012) 46:816–21. 10.1136/bjsports-2012-091395 22875910

[136] Sassano M McKee M Ricciardi W Boccia S Transmission of SARS-CoV-2 and Other Infections at Large Sports Gatherings: A Surprising Gap in Our Knowledge. Front Med (2020) 7:277. 10.3389/fmed.2020.00277 PMC7273227 32574343

[137] Kim TK Lim HR Byun JS Vitamin C supplementation reduces the odds of developing a common cold in Republic of Korea Army recruits: randomised controlled trial. BMJ Mil Heal (2020). 10.1136/bmjmilitary-2019-001384. bmjmilitary-2019-001384. 32139409

[138] Nieman DC Exercise immunology: Nutritional countermeasures. Can J Appl Physiol (2001) 26 Suppl:S45–55. 10.1139/h2001-041 11897882

[139] Powers SK Jackson MJ Exercise-induced oxidative stress: Cellular mechanisms and impact on muscle force production. Physiol Rev (2008) 88:1243–76. 10.1152/physrev.00031.2007 PMC2909187 18923182

[140] Walsh NP Nutrition and Athlete Immune Health: New Perspectives on an Old Paradigm. Sport Med (2019) 49:153–68. 10.1007/s40279-019-01160-3 PMC6901425 31691927

[141] Peternelj TT Coombes JS Antioxidant supplementation during exercise training: Beneficial or detrimental? Sport Med (2011) 41:1043–69. 10.2165/11594400-000000000-00000 22060178

[142] Cobley JN McHardy H Morton JP Nikolaidis MG Close GL Influence of vitamin C and vitamin E on redox signaling: Implications for exercise adaptations. Free Radic Biol Med (2015) 84:65–76. 10.1016/j.freeradbiomed.2015.03.0180 25841784

[143] Margaritelis NV Cobley JN Paschalis V Veskoukis AS Theodorou AA Kyparos A Principles for integrating reactive species into in vivo biological processes: Examples from exercise physiology. Cell Signal (2016) 28:256–71. 10.1016/j.cellsig.2015.12.011 26721187

[144] Merry TL Ristow M Do antioxidant supplements interfere with skeletal muscle adaptation to exercise training? J Physiol (2016) 594:5135–47. 10.1113/JP270654 PMC5023714 26638792

[145] Ismaeel A Holmes M Papoutsi E Panton L Koutakis P Resistance training, antioxidant status, and antioxidant supplementation. Int J Sport Nutr Exerc Metab (2019) 29:539–47. 10.1123/ijsnem.2018-0339 30859847

[146] Righi NC Schuch FB De Nardi AT Pippi CM de Almeida Righi G Puntel GO Effects of vitamin C on oxidative stress, inflammation, muscle soreness, and strength following acute exercise: meta-analyses of randomized clinical trials. Eur J Nutr (2020) 59:2827–39. 10.1007/s00394-020-02215-2 32162041

[147] Rothschild JA Bishop DJ Effects of Dietary Supplements on Adaptations to Endurance Training. Sport Med (2020) 50:25–53. 10.1007/s40279-019-01185-8 31531769

[148] Pastor R Tur JA Antioxidant Supplementation and Adaptive Response to Training: A Systematic Review. Curr Pharm Des (2019) 25:1889–912. 10.2174/1381612825666190701164923 31267859

[149] Mason SA Trewin AJ Parker L Wadley GD Antioxidant supplements and endurance exercise: Current evidence and mechanistic insights. Redox Biol (2020) 35:101471. 10.1016/j.redox.2020.101471 32127289 PMC7284926

[150] Kerksick CM Wilborn CD Roberts MD Smith-Ryan A Kleiner SM Jäger R ISSN exercise & sports nutrition review update: Research & recommendations. J Int Soc Sports Nutr (2018) 15:38. 10.1186/s12970-018-0242-y 30068354 PMC6090881

[151] Paschalis V Theodorou AA Kyparos A Dipla K Zafeiridis A Panayiotou G Low vitamin C values are linked with decreased physical performance and increased oxidative stress: reversal by vitamin C supplementation. Eur J Nutr (2016) 55:45–53. 10.1007/s00394-014-0821-x 25526969

[152] Margaritelis NV Paschalis V Theodorou AA Kyparos A Nikolaidis MG Antioxidants in personalized nutrition and exercise. Adv Nutr (2018) 9:813–23. 10.1093/ADVANCES/NMY052 PMC6247356 30256898

[153] Mandell LA Epidemiology and etiology of community-acquired pneumonia. Infect Dis Clin North Am (2004) 18:761–76. 10.1016/j.idc.2004.08.003 PMC7135665 15555823

[154] Ellison RT Donowitz GR Acute Pneumonia. Mandell, Douglas, and Bennett"s Principles and Practice of Infectious Diseases. (2014). pp. 823–46.e5. 10.1016/B978-1-4557-4801-3.00069-2.

[155] Troeger C Blacker B Khalil IA Rao PC Cao J Zimsen SRM Estimates of the global, regional, and national morbidity, mortality, and aetiologies of lower respiratory infections in 195 countries, 1990–2016: a systematic analysis for the Global Burden of Disease Study 2016. Lancet Infect Dis (2018) 18:1191–210. 10.1016/S1473-3099(18)30310-4 PMC6202443 30243584

[156] Naghavi M Wang H Lozano R Davis A Liang X Zhou M Global, regional, and national age-sex specific all-cause and cause-specific mortality for 240 causes of death, 1990-2013: A systematic analysis for the Global Burden of Disease Study 2013. Lancet (2015) 385:117–71. 10.1016/S0140-6736(14)61682-2 PMC4340604 25530442

[157] Erol N Saglam L Saglam YS Erol HS Altun S Aktas MS The Protection Potential of Antioxidant Vitamins Against Acute Respiratory Distress Syndrome: a Rat Trial. Inflammation (2019) 42:1585–94. 10.1007/s10753-019-01020-2 31081527

[158] Cai Y Li YF Tang LP Tsoi B Chen M Chen H A new mechanism of vitamin C effects on A/FM/1/47(H1N1) virus-induced pneumonia in restraint-stressed mice. BioMed Res Int (2015) 2015:675149. 10.1155/2015/675149 25710018 PMC4331320

[159] Kim WY Hong SB Sepsis and acute respiratory distress syndrome: Recent update. Tuberc Respir Dis (Seoul) (2016) 79:53–7. 10.4046/trd.2016.79.2.53 PMC4823184 27066082

[160] Hemilä H Louhiala P Vitamin C for preventing and treating pneumonia. Cochrane Database Syst Rev (2013) 8:CD005532. 10.1002/14651858.CD005532.pub3 23925826

[161] Padhani Z Moazzam Z Ashraf A Bilal H Salam R Das J Vitamin C supplementation for prevention and treatment of pneumonia. Cochrane Database Syst Rev (2020) 4:CD013134. 10.1002/14651858.CD013134.pub2 32337708 PMC7192369

[162] Chen N Zhou M Dong X Qu J Gong F Han Y Epidemiological and clinical characteristics of 99 cases of 2019 novel coronavirus pneumonia in Wuhan, China: a descriptive study. Lancet (2020) 395:507–13. 10.1016/S0140-6736(20)30211-7 PMC7135076 32007143

[163] Zhu N Zhang D Wang W Li X Yang B Song J A novel coronavirus from patients with pneumonia in China, 2019. N Engl J Med (2020) 382:727–33. 10.1056/NEJMoa2001017 PMC7092803 31978945

[164] Xu Z Shi L Wang Y Zhang J Huang L Zhang C Pathological findings of COVID-19 associated with acute respiratory distress syndrome. Lancet Respir Med (2020) 8:420–2. 10.1016/S2213-2600(20)30076-X PMC7164771 32085846

[165] Pascarella G Strumia A Piliego C Bruno F Del Buono R Costa F COVID-19 diagnosis and management: a comprehensive review. J Intern Med (2020) 288:192–206. 10.1111/joim.13091 32348588 PMC7267177

[166] Iddir M Brito A Dingeo G Fernandez Del Campo S Samouda H La Frano M Strengthening the Immune System and Reducing Inflammation and Oxidative Stress through Diet and Nutrition: Considerations during the COVID-19 Crisis. Nutrients (2020) 12:1562. 10.3390/nu12061562 PMC7352291 32471251

[167] Adams KK Baker WL Sobieraj DM Myth Busters: Dietary Supplements and COVID-19. Ann Pharmacother (2020) 54:820–6. 10.1177/1060028020928052 PMC8685478 32396382

[168] Bauer SR Kapoor A Rath M Thomas SA What is the role of supplementation with ascorbic acid, zinc, vitamin D, or N -acetylcysteine for prevention or treatment of COVID-19? Cleve Clin J Med (2020). 10.3949/ccjm.87a.ccc046 32513807

[169] BourBour F Mirzaei Dahka S Gholamalizadeh M Akbari ME Shadnoush M Haghighi M Nutrients in prevention, treatment, and management of viral infections; special focus on Coronavirus. Arch Physiol Biochem (2020) 1–10. 10.1080/13813455.2020.1791188 32644876

[170] Toresdahl BG Asif IM Coronavirus Disease 2019 (COVID-19): Considerations for the Competitive Athlete. Sport Heal A Multidiscip Approach (2020) 12:221–4. 10.1177/1941738120918876 PMC7222670 32250193

[171] Zabetakis I Lordan R Norton C Tsoupras A COVID-19: The Inflammation Link and the Role of Nutrition in Potential Mitigation. Nutrients (2020) 12:1466. 10.3390/nu12051466 PMC7284818 32438620

[172] Costa FF Rosário WR Ribeiro Farias AC de Souza RG Duarte Gondim RS Barroso WA Metabolic syndrome and COVID-19: An update on the associated comorbidities and proposed therapies. Diabetes Metab Syndr Clin Res Rev (2020) 14:809–14. 10.1016/j.dsx.2020.06.016 PMC7286828 32540733

[173] World Health Organization Information note on COVID-19 and NCDs. (2020)Information note on COVID-19 and NCDs . Available at: https://www.who.int/publications/m/item/covid-19-and-ncds (Accessed July 4, 2020).

[174] Yang J Zheng Y Gou X Pu K Chen Z Guo Q Prevalence of comorbidities and its effects in coronavirus disease 2019 patients: A systematic review and meta-analysis. Int J Infect Dis (2020) 94:91–5. 10.1016/j.ijid.2020.03.017 PMC7194638 32173574

[175] Cai Q Chen F Wang T Luo F Liu X Wu Q Obesity and COVID-19 Severity in a Designated Hospital in Shenzhen, China. Diabetes Care (2020) 43:1392–8. 10.2337/dc20-0576 32409502

[176] Lighter J Phillips M Hochman S Sterling S Johnson D Francois F Obesity in patients younger than 60 years is a risk factor for Covid-19 hospital admission. Clin Infect Dis (2020) 71:896–7. 10.1093/cid/ciaa415 PMC7184372 32271368

[177] Bhasin A Nam H Yeh C Lee J Liebovitz D Achenbach C Is BMI higher in younger patients with COVID-19? Association between BMI and COVID-19 hospitalization by age. Obesity (2020) 28:1811–4. 10.1002/oby.22947 PMC7361943 32610371

[178] Petrilli CM Jones SA Yang J Rajagopalan H O’Donnell L Chernyak Y Factors associated with hospital admission and critical illness among 5279 people with coronavirus disease 2019 in New York City: Prospective cohort study. BMJ (2020) 369:m1966. 10.1136/bmj.m1966 32444366 PMC7243801

[179] Ogden CL Carroll MD Kit BK Flegal KM Prevalence of childhood and adult obesity in the United States, 2011-2012. JAMA - J Am Med Assoc (2014) (219):1–8. 10.1001/jama.2014.732 PMC4770258 24570244

[180] Mah E Matos MD Kawiecki D Ballard K Guo Y Volek JS Vitamin C Status Is Related to Proinflammatory Responses and Impaired Vascular Endothelial Function in Healthy, College-Aged Lean and Obese Men. J Am Diet Assoc (2011) 111:737–43. 10.1016/j.jada.2011.02.003 21515122

[181] Block G Jensen CD Dalvi TB Norkus EP Hudes M Crawford PB Vitamin C treatment reduces elevated C-reactive protein. Free Radic Biol Med (2009) 46:70–7. 10.1016/j.freeradbiomed.2008.09.030 PMC2631578 18952164

[182] Rychter AM Ratajczak AE Zawada A Dobrowolska A Krela-Kaźmierczak I Non-systematic review of diet and nutritional risk factors of cardiovascular disease in obesity. Nutrients (2020) 12:814. 10.3390/nu12030814 PMC7146494 32204478

[183] Rychter AM Zawada A Ratajczak AE Dobrowolska A Krela-Kaźmierczak I Should patients with obesity be more afraid of COVID-19? Obes Rev (2020) 21:e13083. 10.1111/obr.13083 32583537 PMC7362042

[184] Kumar A Arora A Sharma P Anikhindi SA Bansal N Singla V Is diabetes mellitus associated with mortality and severity of COVID-19? A meta-analysis. Diabetes Metab Syndr Clin Res Rev (2020) 14:535–45. 10.1016/j.dsx.2020.04.044 PMC7200339 32408118

[185] International Diabetes Federation. About Diabetes (2020).. Available at: https://idf.org/aboutdiabetes/type-2-diabetes.html (Accessed July 6, 2020).

[186] McArdle MA Finucane OM Connaughton RM McMorrow AM Roche HM Mechanisms of obesity-induced inflammation and insulin resistance: Insights into the emerging role of nutritional strategies. Front Endocrinol (Lausanne) (2013) 4:52. 10.3389/fendo.2013.00052 23675368 PMC3650620

[187] Will JC Byers T Does Diabetes Mellitus Increase the Requirement for Vitamin C? Nutr Rev (2009) 54:193–202. 10.1111/j.1753-4887.1996.tb03932.x 8918139

[188] Sargeant LA Wareham NJ Bingham S Day NE Luben RN Oakes S Vitamin C and hyperglycemia in the European Prospective Investigation Into Cancer - Norfolk (EPIC-Norfolk) study. Diabetes Care (2000) 23:726–32. 10.2337/diacare.23.6.726 10840986

[189] Sinclair AJ Taylor PB Lunec J Girling AJ Barnett AH Low Plasma Ascorbate Levels in Patients with Type 2 Diabetes Mellitus Consuming Adequate Dietary Vitamin C. Diabetes Med (1994) 11:893–8. 10.1111/j.1464-5491.1994.tb00375.x 7705029

[190] Wilson R Willis J Gearry R Skidmore P Fleming E Frampton C Inadequate vitamin C status in prediabetes and type 2 diabetes mellitus: Associations with glycaemic control, obesity, and smoking. Nutrients (2017) 9:997. 10.3390/nu9090997 PMC5622757 28891932

[191] Seghieri G Martinoli L Miceli M Ciuti M D’Alessandri G Gironi A Renal excretion of ascorbic acid in insulin dependent diabetes mellitus. Int J Vitam Nutr Res (1994) 64:119–24. 7960490

[192] Luc K Schramm-Luc A Guzik TJ Mikolajczyk TP Oxidative stress and inflammatory markers in prediabetes and diabetes. J Physiol Pharmacol (2019) 70(6). 10.26402/jpp.2019.6.01 32084643

[193] Mason SA Della Gatta PA Snow RJ Russell AP Wadley GD Ascorbic acid supplementation improves skeletal muscle oxidative stress and insulin sensitivity in people with type 2 diabetes: Findings of a randomized controlled study. Free Radic Biol Med (2016) 93:227–38. 10.1016/j.freeradbiomed.2016.01.006 26774673

[194] Ellulu MS Rahmat A Ismail P Khaza’ai H Abed Y Effect of vitamin C on inflammation and metabolic markers in hypertensive and/or diabetic obese adults: a randomized controlled trial. Drug Des Devel Ther (2015) 9:3405–12. 10.2147/DDDT.S83144 PMC4492638 26170625

[195] Ashor AW Werner AD Lara J Willis ND Mathers JC Siervo M Effects of Vitamin C supplementation on glycaemic control: A systematic review and meta-analysis of randomised controlled trials. Eur J Clin Nutr (2017) 71:1371–80. 10.1038/ejcn.2017.24 28294172

[196] Fang L Karakiulakis G Roth M Are patients with hypertension and diabetes mellitus at increased risk for COVID-19 infection? Lancet Respir Med (2020) 8:e21. 10.1016/S2213-2600(20)30116-8 32171062 PMC7118626

[197] Zhou F Yu T Du R Fan G Liu Y Liu Z Clinical course and risk factors for mortality of adult inpatients with COVID-19 in Wuhan, China: a retrospective cohort study. Lancet (2020) 395:1054–62. 10.1016/S0140-6736(20)30566-3 PMC7270627 32171076

[198] Zheng Z Peng F Xu B Zhao J Liu H Peng J Risk factors of critical & mortal COVID-19 cases: A systematic literature review and meta-analysis. J Infect (2020) 81:e16–25. 10.1016/j.jinf.2020.04.021 PMC7177098 32335169

[199] Gheblawi M Wang K Viveiros A Nguyen Q Zhong J-C Turner AJ Angiotensin-Converting Enzyme 2: SARS-CoV-2 Receptor and Regulator of the Renin-Angiotensin System. Circ Res (2020) 126:1456–74. 10.1161/circresaha.120.317015 PMC7188049 32264791

[200] Chen L Hao G The role of angiotensin-converting enzyme 2 in coronaviruses/influenza viruses and cardiovascular disease. Cardiovasc Res (2020) 116:1932–6. 10.1093/cvr/cvaa093 PMC7184394 32267499

[201] Touyz RM Li H Delles C ACE2 the Janus-faced protein – from cardiovascular protection to severe acute respiratory syndrome-coronavirus and COVID-19. Clin Sci (2020) 134:747–50. 10.1042/CS20200363 32255491

[202] Tan HW Xu Y Lau ATY Angiotensin-converting enzyme 2: The old door for new severe acute respiratory syndrome coronavirus 2 infection. Rev Med Virol (2020) 30:e2122. 10.1002/rmv.2122 32602627 PMC7361198

[203] Khaw KT Bingham S Welch A Luben R Wareham N Oakes S Relation between plasma ascorbic acid and mortality in men and women in EPIC-Norfolk prospective study: A prospective population study. Lancet (2001) 357:657–63. 10.1016/S0140-6736(00)04128-3 11247548

[204] Pfister R Sharp SJ Luben R Wareham NJ Khaw KT Plasma vitamin C predicts incident heart failure in men and women in European Prospective Investigation into Cancer and Nutrition-Norfolk prospective study. Am Heart J (2011) 162:246–53. 10.1016/j.ahj.2011.05.007 21835284

[205] Ingles DP Cruz Rodriguez JB Garcia H Supplemental Vitamins and Minerals for Cardiovascular Disease Prevention and Treatment. Curr Cardiol Rep (2020) 22:22. 10.1007/s11886-020-1270-1 32067177

[206] Moser MA Chun OK Vitamin C and heart health: A review based on findings from epidemiologic studies. Int J Mol Sci (2016) 17:1328. 10.3390/ijms17081328 PMC5000725 27529239

[207] Lykkesfeldt J On the effect of vitamin C intake on human health: How to (mis)interprete the clinical evidence. Redox Biol (2020) 34:101532. 10.1016/j.redox.2020.101532 32535545 PMC7296342

[208] Chow N Fleming-Dutra K Gierke R Hall A Hughes M Pilishvili T Preliminary Estimates of the Prevalence of Selected Underlying Health Conditions Among Patients with Coronavirus Disease 2019 — United States, February 12–March 28, 2020. MMWR Morb Mortal Wkly Rep (2020) 69:382–6. 10.15585/mmwr.mm6913e2 PMC7119513 32240123

[209] Wu C Chen X Cai Y Xia J Zhou X Xu S Risk Factors Associated with Acute Respiratory Distress Syndrome and Death in Patients with Coronavirus Disease 2019 Pneumonia in Wuhan, China. JAMA Intern Med (2020) 180:934–43. 10.1001/jamainternmed.2020.0994 PMC7070509 32167524

[210] Pae M Meydani SN Wu D The role of nutrition in enhancing immunity in aging. Aging Dis (2012) 3:91–129. 22500273 PMC3320807

[211] Meyer KC The role of immunity and inflammation in lung senescence and susceptibility to infection in the elderly. Semin Respir Crit Care Med (2010) 31:561–74. 10.1055/s-0030-1265897 20941657

[212] Volkert D Visser M Corish CA Geisler C de Groot L Cruz-Jentoft AJ Joint action malnutrition in the elderly (MaNuEL) knowledge hub: summary of project findings. Eur Geriatr Med (2020) 11:169–77. 10.1007/s41999-019-00264-3 32297234

[213] PowerSEJefferyIBRossRPStantonCO’ToolePWO’ConnorEM Food and nutrient intake of irish community-dwelling elderly subjects: Who is at nutritional risk? J Nutr Heal Aging (2014) 18:561–72. 10.1007/s12603-014-0449-9 24950145

[214] Haase H Rink L The immune system and the impact of zinc during aging. Immun Ageing (2009) 6:9. 10.1186/1742-4933-6-9 19523191 PMC2702361

[215] Hunt C Chakravorty NK Annan G Habibzadeh N Schorah CJ The clinical effects of vitamin C supplementation in elderly hospitalised patients with acute respiratory infections. Int J Vitam Nutr Res (1994) 64:212–9. 7814237

[216] Fletcher AE Breeze E Shetty PS Antioxidant vitamins and mortality in older persons: Findings from the nutrition add-on study to the Medical Research Council Trial of Assessment and Management of Older People in the Community. Am J Clin Nutr (2003) 78:999–1010. 10.1093/ajcn/78.5.999 14594788

[217] Yanase F Fujii T Naorungroj T Belletti A Luethi N Carr AC Harm of IV High-Dose Vitamin C Therapy in Adult Patients. Crit Care Med (2020) 48:e620–8. 10.1097/CCM.0000000000004396 32404636

[218] Traxer O Huet B Poindexter J Pak CYC Pearle MS Effect of ascorbic acid consumption on urinary stone risk factors. J Urol (2003) 170:397–401. 10.1097/01.ju.0000076001.21606.53 12853784

[219] Massey LK Liebman M Kynast-Gales SA Ascorbate Increases Human Oxaluria and Kidney Stone Risk. J Nutr (2005) 135:1673–7. 10.1093/jn/135.7.1673 15987848

[220] Ferraro PM Curhan GC Gambaro G Taylor EN Total, dietary, and supplemental Vitamin C intake and risk of incident kidney stones. Am J Kidney Dis (2016) 67:400–7. 10.1053/j.ajkd.2015.09.005 PMC4769668 26463139

[221] Padayatty SJ Sun H Wang Y Riordan HD Hewitt SM Katz A Vitamin C Pharmacokinetics: Implications for Oral and Intravenous Use. Ann Intern Med (2004) 140:533–7. 10.7326/0003-4819-140-7-200404060-00010 15068981

[222] Galley HF Davies MJ Webster NR Ascorbyl radical formation in patients with sepsis: Effect of ascorbate loading. Free Radic Biol Med (1996) 20:139–43. 10.1016/0891-5849(95)02022-5 8903690

[223] Schorah CJ Downing C Piripitsi A Gallivan L Al-Hazaa AH Sanderson MJ Total vitamin C, ascorbic acid, and dehydroascorbic acid concentrations in plasma of critically ill patients. Am J Clin Nutr (1996) 63:760–5. 10.1093/ajcn/63.5.760 8615361

[224] Metnitz PGH Bartens C Fischer M Fridrich P Steltzer H Druml W Antioxidant status in patients with acute respiratory distress syndrome. Intensive Care Med (1999) 25:180–5. 10.1007/s001340050813 10193545

[225] Kieffer P Thannberger P Wilhelm JM Kieffer C Schneider F Multiple organ dysfunction dramatically improving with the infusion of vitamin C: More support for the persistence of scurvy in our “welfare” society. Intensive Care Med (2001) 27:448. 10.1007/s001340000830 11396297

[226] Long CL Maull KI Krishnan RS Laws HL Geiger JW Borghesi L Ascorbic acid dynamics in the seriously ill and injured. J Surg Res (2003) 109:144–8. 10.1016/S0022-4804(02)00083-5 12643856

[227] Fowler AA Syed AA Knowlson S Sculthorpe R Farthing D DeWilde C Phase I safety trial of intravenous ascorbic acid in patients with severe sepsis. J Transl Med (2014) 12:32. 10.1186/1479-5876-12-32 24484547 PMC3937164

[228] Carr AC Spencer E Dixon L Chambers ST Patients with community acquired pneumonia exhibit depleted vitamin c status and elevated oxidative stress. Nutrients (2020) 12:1318. 10.3390/nu12051318 PMC7284353 32384616

[229] Oudemans-van Straaten HM Spoelstra-de Man AME de Waard MC Vitamin C revisited. Crit Care (2014) 18:460. 10.1186/s13054-014-0460-x 25185110 PMC4423646

[230] Seno T Inoue N Matsui K Ejiri J Hirata KI Kawashima S Functional expression of sodium-dependent vitamin C transporter 2 in human endothelial cells. J Vasc Res (2004) 41:345–51. 10.1159/000080525 15340249

[231] Schmidt R Luboeinski T Markart P Ruppert C Daum C Grimminger F Alveolar antioxidant status in patients with acute respiratory distress syndrome. Eur Respir J (2004) 24:994–9. 10.1183/09031936.04.00120703 15572544

[232] Banerjee D Kaul D Combined inhalational and oral supplementation of ascorbic acid may prevent influenza pandemic emergency: A hypothesis. Nutrition (2010) 26:128–32. 10.1016/j.nut.2009.09.015 PMC7127226 20005468

[233] Barnes BJ Adrover JM Baxter-Stoltzfus A Borczuk A Cools-Lartigue J Crawford JM Targeting potential drivers of COVID-19: Neutrophil extracellular traps. J Exp Med (2020) 217:e20200652. 10.1084/jem.20200652 32302401 PMC7161085

[234] McGonagle D Sharif K O’Regan A Bridgewood C The Role of Cytokines including Interleukin-6 in COVID-19 induced Pneumonia and Macrophage Activation Syndrome-Like Disease. Autoimmun Rev (2020) 19:102537. 10.1016/j.autrev.2020.102537 32251717 PMC7195002

[235] Pierrakos C Karanikolas M Scolletta S Karamouzos V Velissaris D Acute respiratory distress syndrome: pathophysiology and therapeutic options. J Clin Med Res (2012) 4:7–16. 10.4021/jocmr761w 22383921 PMC3279495

[236] Chen Y Luo G Yuan J Wang Y Yang X Wang X Vitamin C Mitigates Oxidative Stress and Tumor Necrosis Factor-Alpha in Severe Community-Acquired Pneumonia and LPS-Induced Macrophages. Mediators Inflammation (2014) 2014:426740. 10.1155/2014/426740 PMC4165740 25253919

[237] Härtel C Strunk T Bucsky P Schultz C Effects of vitamin C on intracytoplasmic cytokine production in human whole blood monocytes and lymphocytes. Cytokine (2004) 27:101–6. 10.1016/j.cyto.2004.02.004 15271375

[238] Fisher BJ Seropian IM Kraskauskas D Thakkar JN Voelkel NF Fowler AA Ascorbic acid attenuates lipopolysaccharide-induced acute lung injury. Crit Care Med (2011) 39:1454–60. 10.1097/CCM.0b013e3182120cb8 21358394

[239] Fisher BJ Kraskauskas D Martin EJ Farkas D Wegelin JA Brophy D Mechanisms of attenuation of abdominal sepsis induced acute lung injury by ascorbic acid. Am J Physiol - Lung Cell Mol Physiol (2012) 303:L20–32. 10.1152/ajplung.00300.2011 22523283

[240] Molina N Morandi AC Bolin AP Otton R Comparative effect of fucoxanthin and vitamin C on oxidative and functional parameters of human lymphocytes. Int Immunopharmacol (2014) 22:41–50. 10.1016/j.intimp.2014.06.026 24975831

[241] Zhang XY Xu ZP Wang W Cao JB Fu Q Zhao WX Vitamin C alleviates LPS-induced cognitive impairment in mice by suppressing neuroinflammation and oxidative stress. Int Immunopharmacol (2018) 65:438–47. 10.1016/j.intimp.2018.10.020 30388518

[242] Fowler AA Truwit JD Hite RD Morris PE Dewilde C Priday A Effect of Vitamin C Infusion on Organ Failure and Biomarkers of Inflammation and Vascular Injury in Patients with Sepsis and Severe Acute Respiratory Failure: The CITRIS-ALI Randomized Clinical Trial. JAMA (2019) 322:1261–70. 10.1001/jama.2019.11825 PMC6777268 31573637

[243] Marik PE Payen D CITRIS-ALI: How statistics were used to obfuscate the true findings. Anaesth Crit Care Pain Med (2019) 38:575–7. 10.1016/j.accpm.2019.10.004 31785700

[244] Zhang M Jativa DF Vitamin C supplementation in the critically ill: A systematic review and meta-analysis. SAGE Open Med (2018) 6:2050312118807615. 10.1177/2050312118807615 30364374 PMC6196621

[245] Langlois PL Manzanares W Adhikari NKJ Lamontagne F Stoppe C Hill A Vitamin C Administration to the Critically Ill: A Systematic Review and Meta-Analysis. JPEN J Parenter Enteral Nutr (2019) 43:335–46. 10.1002/jpen.1471 30452091

[246] Hemilä H Chalker E Vitamin C may reduce the duration of mechanical ventilation in critically ill patients: A meta-regression analysis. J Intensive Care (2020) 8:15. 10.1186/s40560-020-0432-y 32047636 PMC7006137

[247] Zabet M Mohammadi M Ramezani M Khalili H Effect of high-dose Ascorbic acid on vasopressor′s requirement in septic shock. J Res Pharm Pract (2016) 5:94–100. 10.4103/2279-042x.179569 27162802 PMC4843590

[248] Wei X Wang Z Liao X Guo W Wen JY Qin T Efficacy of vitamin C in patients with sepsis: An updated meta-analysis. Eur J Pharmacol (2020) 868:172889. 10.1016/j.ejphar.2019.172889 31870831

[249] Kim WY Jo EJ Eom JS Mok J Kim MH Kim KU hydrocortisone, and thiamine therapy for patients with severe pneumonia who were admitted to the intensive care unit: Propensity score-based analysis of a before-after cohort study. J Crit Care (2018) 47:211–8. 10.1016/j.jcrc.2018.07.004 30029205

[250] Marik PE Khangoora V Rivera R Hooper MH Catravas J Hydrocortisone, Vitamin C, and Thiamine for the Treatment of Severe Sepsis and Septic Shock: A Retrospective Before-After Study. Chest (2017) 151:1229–38. 10.1016/j.chest.2016.11.036 27940189

[251] Fujii T Luethi N Young PJ Frei DR Eastwood GM French CJ Effect of Vitamin C, Hydrocortisone, and Thiamine vs Hydrocortisone Alone on Time Alive and Free of Vasopressor Support among Patients with Septic Shock: The VITAMINS Randomized Clinical Trial. JAMA (2020) 323:423–31. 10.1001/jama.2019.22176 PMC7029761 31950979

[252] Carr AC Is the VITAMINS RCT indicating potential redundancy between corticosteroids and vitamin C? Crit Care (2020) 24:129. 10.1186/s13054-020-02853-2 32252799 PMC7137516

[253] Frommelt MA Kory P Long MT Letter on Update to the Vitamin C, Thiamine, and Steroids in Sepsis (VICTAS) Protocol. Trials (2020) 21:350. 10.1186/s13063-020-04289-z 32317008 PMC7175527

[254] Carr AC Vitamin C in Pneumonia and Sepsis. In: Chen Q Vissers MCM, editors. Vitamin C: New Biochemical and Functional Insights. Boca Raton, FL: CRC Press (2020). p. 115–35. 10.1201/9780429442025-7 33750090

[255] Romano S Galante H Figueira D Mendes Z Rodrigues AT Time-trend analysis of medicine sales and shortages during COVID-19 outbreak: Data from community pharmacies. Res Soc Adm Pharm (2020) 1551-7411(20):30611–2. 10.1016/j.sapharm.2020.05.024 PMC7245321 32482587

